# The genetics of adaptation in freshwater Eurasian shad (*Alosa*)

**DOI:** 10.1002/ece3.8908

**Published:** 2022-05-24

**Authors:** Stephen J. Sabatino, Paulo Pereira, Miguel Carneiro, Jolita Dilytė, John Patrick Archer, Antonio Munoz, Francesco Nonnis‐Marzano, Antonio Murias

**Affiliations:** ^1^ CIBIO ‐ Centro de Investigação em Biodiversidade e Recursos Genéticos Universidade do Porto Vairão Portugal; ^2^ BIOPOLIS ‐ Program in Genomics, Biodiversity and Land Planning CIBIO Vairão Portugal; ^3^ Departamento de Biologia Faculdade de Ciências Universidade do Porto Porto Portugal; ^4^ Department of Chemistry, Life Sciences and Environmental Sustainability Università di Parma Parma Italy

**Keywords:** adaptation, genomics, parallel evolution, phenotypic convergence, whole‐genome pool‐seq

## Abstract

Studying the genetics of phenotypic convergence can yield important insights into adaptive evolution. Here, we conducted a comparative genomic study of four lineages (species and subspecies) of anadromous shad (*Alosa*) that have independently evolved life cycles entirely completed in freshwater. Three naturally diverged (*A*. *fallax lacustris*, *A*.* f*. *killarnensis*, and *A*.* macedonica*), and the fourth (*A*. *alosa*) was artificially landlocked during the last century. To conduct this analysis, we assembled and annotated a draft of the *A*. *alosa* genome and generated whole‐genome sequencing for 16 anadromous and freshwater populations of shad. Widespread evidence for parallel genetic changes in freshwater populations within lineages was found. In freshwater *A*. *alosa*, which have only been diverging for tens of generations, this shows that parallel adaptive evolution can rapidly occur. However, parallel genetic changes across lineages were comparatively rare. The degree of genetic parallelism was not strongly related to the number of shared polymorphisms between lineages, thus suggesting that other factors such as divergence among ancestral populations or environmental variation may influence genetic parallelism across these lineages. These overall patterns were exemplified by genetic differentiation involving a paralog of *ATPase*‐*α1* that appears to be under selection in just two of the more distantly related lineages studied, *A*.* f*. *lacustris* and *A*. *alosa*. Our findings provide insights into the genetic architecture of adaptation and parallel evolution along a continuum of population divergence.

## Graphical abstract

Here, we studied the genomics of adaptation in anadromous shad that have evolved a completely freshwater life cycle. This included conducting genome scans with a de novo genome assembly and pool‐seq data for 16 populations of shad. We used these data to study patterns of parallel genetic evolution within and across the four lineages examined.

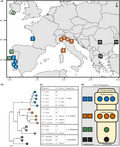

## 1 INTRODUCTION

Organisms often evolve similar phenotypes to adapt to ecological changes or expand their niches (Endler, 1986). When such phenotypic convergence occurs, analogous selective forces are often involved. However, the extent to which identical, or related genetic alternations (i.e., “parallel”) are responsible for phenotypic convergence varies greatly both within and across taxa (Bailey et al., 2015; Oke et al., 2017; Perrier et al., 2013; Stern, 2013). While our understanding of parallelism has progressed in recent years, a general understanding of the ecological and evolutionary circumstances initiating its occurrence remains elusive (Losos, 2011; Stern, 2013; Storz, 2016).

The likelihood of parallel evolution can depend on several factors. In the absence of gene flow, unless populations share advantageous polymorphisms, parallelism is dependent upon the same, or similar, alleles arising via de novo mutation within each newly colonized location or ecological setting (Colosimo et al., 2005). This can take hundreds or thousands of generations in higher organisms such as vertebrates (Hoban et al., 2016). Thus, selection upon existing shared polymorphism is usually the most likely way for parallelism to arise. This hypothesis has been supported by studies on a variety of species, including stickleback (Colosimo et al., 2005; Nelson & Cresko, 2018; Oke et al., 2017), cod (Bradbury et al., 2010), and herring (Lamichhaney et al., 2017). However, since ecological adaptation involves an ecological shift, alleles for relevant fitness‐related traits may be slightly deleterious, or under balancing selection, within the ancestral setting. Consequently, such alleles will tend to segregate at low or intermediate frequencies in ancestral lineages (Chevin et al., 2010). This lowers the probability that they will be present in newly founded populations, and when existent, it increases the chance that they will be lost due to genetic drift.

Gene flow can promote parallelism by increasing levels of shared polymorphism (Stuart et al., 2017). However, gene flow can also have mixed effects on the rate and direction of adaptive divergence. The ultimate consequences of gene flow may depend on factors including the intensity and consistency of selection, the genetic architecture of the trait (Garcia‐Ramos & Kirkpatrick, 1997; Star & Spencer, 2013), and interactions among the genes involved (Chevin et al., 2010). In addition, when multiple alleles with similar phenotypic effects are present, the likelihood of nonparallel evolution can increase by chance alone (Stern, 2013), or for other reasons such as subtle differences in spatially varying selection pressure (Conte et al., 2012; Frickel et al., 2018). Indeed, selection due to environmental variation has been found to influence parallelism in several species including stickleback (Hohenlohe et al., 2010), cichlid fish (Elmer et al., 2014), whitefish (Gagnaire et al., 2013), stick insects (Soria‐Carrasco et al., 2014), stickleback (Raeymaekers et al., 2017; Stuart et al., 2017), and marine snails (Morales et al., 2019). Due to the range of factors that can affect the likelihood of parallelism, it is now widely accepted that it will only occur in specific circumstances.

**FIGURE 1:**
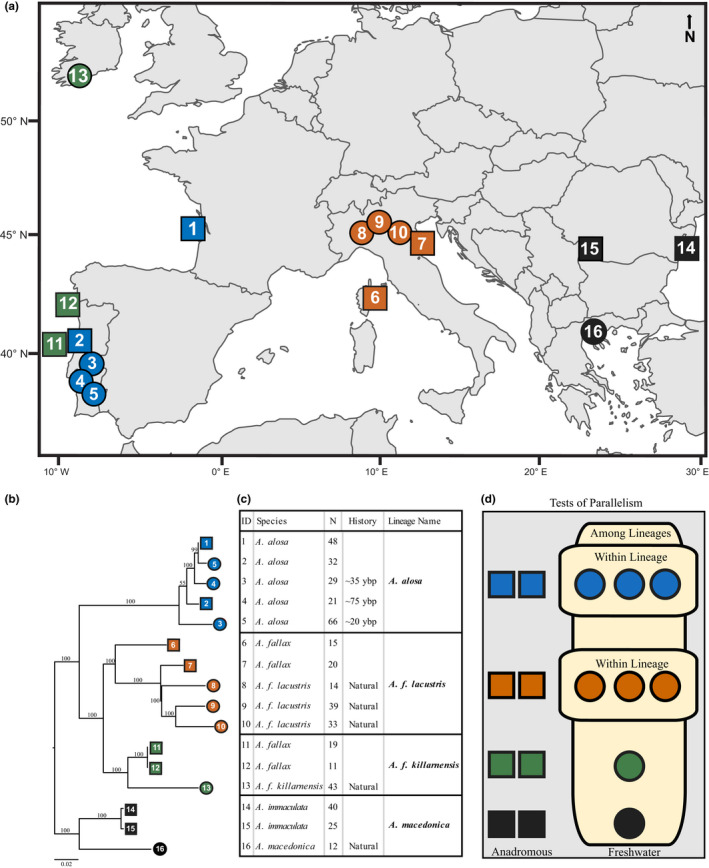
(a) Map of sampling locations for the four lineages studied: *A*.* alosa*—Atlantic (navy); *A*.* f*. *lacustris*—Italy (orange); *A*.* f*. *killarnensis*—Ireland (green); and *A*.* macedonica*—Black Sea (black). The name of each location sampled is as follows: 1—Garonne River; 2—Mondego River; 3—Aguieira Reservoir; 4—Castelo de Bode Reservoir; 5—Alqueva Reservoir; 6—Tavignano River; 7—Po River; 8—Lake Maggiore; 9—Lake Como; 10—Lake Garda; 11—Mondego River; 12—Lima River; 13—Lough Leane; 14—Danube River, Tulcea; 15—Danube River, Iron Gate; and 16—Lake Volvi. The locations marked by a square on the map are anadromous populations, and those with a circle are freshwater. (b) Phylogenetic tree of the populations studied based on genetic distance (*F*_ST_). (c) The map identification number (ID), species, sample size, and population history of each sample. (d) A schematic of the experimental design used in this study

To further our understanding of these topics, we studied the genetics of adaptation in four independent lineages of Eurasian shad (alosines; genus *Alosa*) (Linck, 1790) that are typically anadromous, but also include populations with a completely freshwater life history (Figure 1, Table S1). These alosines are an extraordinary model system for studying parallelism for several reasons. First, phenotypic differences between freshwater and anadromous shad are usually similar and involve traits known to have a genetic basis in other fish species, including size, parity, and numbers of gill rakers (Aprahamian et al., 2003). Second, having diverged from each other <1.5 million years ago, the Eurasian shad are closely related lineages (Faria et al., 2012). Lastly, the alosine model enables us to study the genetics of adaptation in populations that have diverged in different ways. For example, while the freshwater *A*.* alosa* we studied were all artificially landlocked in reservoirs in Portugal during the last century and evolved without gene flow (Aprahamian et al., 2003; Pereira et al., 1999), the freshwater populations of three of the lineages (species and subspecies) we examined inhabit lakes in Italy (*A*.* f*.* lacustris*), Ireland (*A*.* f*.* killarnensis*), and Greece (*A*.* macedonica*) and naturally diverged sometimes during the last 30 thousand years after the retreat of the last Pleistocene glaciers (Coscia et al., 2013; Faria et al., 2012). The phenotypic changes observed in these freshwater shad appear to reflect this difference in divergence time. Specifically, while freshwater shad in all four lineages have experienced a sharp decrease in body size (Aprahamian et al., 2003; Pereira et al., 1999), it has been least dramatic in *A*.* alosa*. Additionally, within *A*.* alosa*, individuals belonging to the oldest freshwater population (~75 years) are the smallest of the three lineages that exist, suggesting the adaptive process is ongoing in this lineage. Studying the evolution of these freshwater shads is therefore an excellent opportunity to gain insight into the factors that influence parallelism including the phylogenetic and temporal scales at which it occurs and the impacts of gene flow.

In this study, we generated a de novo genome assembly for *A*.* alosa* and conducted whole‐genome resequencing to identify loci that may be under natural selection in freshwater populations of shad. The resulting data were then used to determine whether parallel genetic evolution may have occurred within or across the lineages studied. Here, “parallel evolution” or “parallelism” refers to allele frequency changes in the same gene or genomic region, but not necessarily the same mutation. Loci involved in adaptation to freshwater in these lineages were identified by searching for genomic regions with extraordinary differences in allele frequencies between anadromous and freshwater populations. Such genome scans have yielded some of the most influential insights into the genetic architecture of adaptation and speciation (Alves et al., 2019; Cresko et al., 2004; Jones et al., 2012; Losos, 1998; Nachman et al., 2003; Vitti et al., 2013). However, since neutral genetic changes can result in patterns of allele frequencies that are similar to those caused by natural selection (Ralph & Coop, 2010), genome scans can yield false‐positive results. To help avoid this pitfall, we separately analyzed each lineage. Given what is known about rapid evolution and adaptation to freshwater in other anadromous species, we expected that parallelism would be positively related to the amount of polymorphism shared between lineages, and the lowest in *A*.* alosa*.

## 2 MATERIALS AND METHODS

### 2.1 Genome assembly and annotation

Blood and muscle tissues were taken from a single freshwater *A*.* alosa* caught in the Aguieira Reservoir, Portugal, by a local fisherman and stored on ice for several hours until it could be placed at −80°C for long‐term storage. DNA was extracted from blood or tissue from the specimen caught using EasySpin^TM^ 96‐well extraction plates according to the manufacturer’s recommendations except that vortexing the DNA was avoided to minimize shearing. DNA from multiple extractions was concentrated to satisfy the requirements of the following sequencing library methods.

The Allis shad (*A*.* alosa*) genome was assembled using a combination of data from short‐read and mate‐pair sequencing libraries. For contig building, one 250‐bp insert library with overlapping reads (2×150 bp paired‐end) was generated using Illumina paired‐end chemistry (V2) and run on three lanes of an Illumina MiSeq 2500. Scaffolding was accomplished by mate‐pair libraries (insert sizes 3 kilobases (kb), 5 kb, 7 kb, and 10 kb) made using the Nextera Mate‐Pair Library Preparation Kit (Illumina). The mate‐pair libraries were multiplexed and run together on three lanes of an Illumina HiSeq 1500. In addition, a 20‐kb mate‐pair dataset was generated by Lucigen Corporation. The *A*.* alosa* genome was assembled using ALLPATHS‐LG‐52488 (Butler et al., 2008). ALLPATHS‐LG‐52488 was run with default settings with the exception that the HAPLOIDIFY option was engaged. To determine the optimal coverage of short‐read, overlapping sequence data for the Allis genome assembly using ALLPATHS‐LG‐52488, the assembly was run using from 40X to 60X coverage at 5X increments, and the coverage that resulted in the greatest contig N50, 55X, was used for the final assembly.

The genome assembly was annotated by analysis of sequence composition and by generating ab initio gene models using transcriptome and protein data. All software for the annotation was run using default parameters unless otherwise specified. Repeat regions were identified and masked using RepeatMasker (Tarailo‐Graovac & Chen, 2009) with an *A*.* alosa*‐specific repeat library that was generated for our de novo shad genome using RepeatModeler (http://www.repeatmasker.org/RepeatModeler/) and RepBase23.02. Intron/exon boundaries were inferred by aligning RNA‐seq data generated from liver, muscle, and gill tissue types (see Supplemental Methods) to our shad genome using Hisat2 (Kim et al., 2019). The aligned RNA reads were used to obtain a genome‐guided transcriptome assembly using Cufflinks (v2.2.1) (Trapnell et al., 2012). An initial run of Maker2 (Holt & Yandell, 2011) was conducted on the repeat‐masked genome using the output from Cufflinks, a publicly available transcriptome assembly for *A*.* alosa* based on RNA‐seq data from ten tissue types (Pasquier et al., 2016) and high‐confidence protein sequence evidence from the UniProt Swiss‐Prot and UniProt *Danio reiro* databases. The genome was then re‐annotated with *Maker2* using gene models generated with GeneMark‐ES (Lomsadze, 2005). To characterize the biological functions of the expected transcripts of each gene model and retrieve PFAM and GO terms, we used Sma3s (Casimiro‐Soriguer et al., 2017). Transcripts were also compared with the UniProt protein databases mentioned earlier using BLASTp (v2.2.28+), and tRNAs were identified using tRNAscan‐SE‐2.0 (Lowe & Eddy, 1997). Finally, SnpEff (v.3.4; Cingolani et al., 2014) was used to annotate variants and classify them as nonsynonymous, synonymous, UTR, 5 kb upstream, 5 kb downstream, intronic, or intergenic. Assembly and annotation completeness was quantified by the number of conserved single‐copy orthologs present both in the reference sequence and in the annotation using the Benchmarking Universal Single‐Copy Orthologs (BUSCO) software (v. 3) with the Actinopterygii_odb9 standard dataset (Simão et al., 2015).

### 2.2 Allele frequencies of SNP loci

Allele frequencies of SNP loci for use in population analysis and genome scans were estimated using a pooled DNA sequencing approach. Muscle, fin, liver, or blood was taken from 467 adult anadromous and freshwater shad caught in each location by commercial fishermen or angling from 1991 to 2015 (Figure 1; Table S1). All anadromous individuals sampled were caught in rivers or streams as they migrated upstream to spawn. Tissue samples were preserved in 95% ethanol or placed on ice and then stored at −80°C. DNA was extracted from tissue samples using EasySpin^TM^ 96‐well extraction plates according to the manufacturer’s recommendations. Eurasian shad can naturally hybridize (Aprahamian et al., 2003; Taillebois et al., 2019). To avoid including any hybrids in the pools, the individuals used for them were prescreened using microsatellite markers as described in Sabatino et al. (2022). For each of the 16 sampling locations, pooled DNA samples were made from 100 ng of DNA per individual. Each of the 16 pools was then tagged with a unique Nextera sequence tag and prepared for sequencing using paired‐end (2 × 125 bp) Illumina chemistry. The pooled DNA libraries were sequenced on an Illumina HiSeq 1500 to an effective coverage from 24× to 46× per pool with an average of 32× (Table S1). The resulting reads were trimmed with Trimmomatic version 0.32 (Bolger et al., 2014) with the following settings: illuminaclip:Tru‐Seq2‐PE.fa:2:30:10 slidingwindow:4:20 leading:10 trailing:10 crop:101 headcrop:0 minlen:80. FASTQC v10.1 (Babraham Bioinformatics) was then run to check the data overall for base and sequence quality.

The pool‐seq data were then used to estimate allele frequencies for SNP loci in each of the four lineages (Figure 1) studied. First, the trimmed pool‐seq datasets were mapped to our *A*.* alosa* genome assembly using BWA‐MEM (Li & Durbin, 2009) with default settings, followed by local realignment performed using GATK RealignerTargetCreator and IndelRealigner options (McKenna et al., 2010). An initial set of DNA sequence variants was then identified using Samtools mpileup (Li et al., 2009). The set of variants identified using Samtools was filtered with the Perl script mpileup2sync.pl of Popoolation2 (Kofler et al., 2011) with the following settings: base quality (Q20), minimum count of the minor allele set to four, minimum coverage of 10, and maximum coverage set at twice the genome‐wide average (per population). The resulting set of SNP loci was further filtered by removing those not identified as biallelic variants, and retaining only SNPs with an overall quality score >20 using FreeBayes v0.9.21 (Garrison & Marth, 2012) with the following set of parameters: use‐best‐n‐alleles 4, pooled‐discrete, min‐coverage 10, min‐repeat‐entropy 1, min‐mapping‐quality 60, min‐base‐quality 20, use‐mapping‐quality, and max‐coverage 100. FreeBayes was run separately for each of the four lineages studied (Figure 1; Table S1). In addition, SNP loci located within 10 bp from an insertion or deletion as identified by FreeBayes were filtered from the final dataset. Allele frequencies for the filtered set of SNP loci were then calculated for each population using Popoolation2 with “pool size” set to twice the number of individuals in each pool as suggested for diploid organisms.

### 2.3 Genome scans for loci under selection

Loci that may have been influenced by natural selection were identified using a sliding window analysis of the change in allele frequencies of SNP loci (ΔAF) between anadromous and freshwater populations within each of the four lineages described in the previous section. To reduce the effect of population history, ΔAF estimates were standardized by subtracting each from the genome‐wide mean (per lineage) and then dividing by the standard deviation. Allele frequency differences between all possible pairs of anadromous and freshwater populations within each lineage were then averaged. Finally, a sliding window approach was used where genomic windows were defined as the average ΔAF for 20 consecutive SNP loci along each scaffold and calculated every 10 SNPs (i.e., step size). To avoid high error rates associated with smaller scaffolds enriched with repetitive sequences, windows were only calculated for the largest 1000 scaffolds, which physically covered 813Mb or 91% of the *A*.* alosa* genome.

The statistical significance of extreme values of average ΔAF in each lineage was then evaluated using permutations. In each permutation, populations were randomly assigned as anadromous or landlocked, and then, the ΔAF for each SNP window was calculated as described earlier with the exception that the starting location to begin counting consecutive SNPs was randomly chosen to range from 1 to 20, which allows for all possible window combinations of 20‐SNP windows to be sampled. A thousand permutations were conducted per lineage. Significant ΔAF can be a consequence of divergent selection or genetic hitchhiking (Lewontin & Krakauer, 1973). Since the freshwater populations studied have been diverging for different lengths of time (tens to thousands of generations), and for comparative purposes, we conducted permutation tests to identify outliers using two different thresholds (99th and 99.9th percentiles). We considered genomic windows with ΔAF in the 99th and 99.9th percentiles (i.e., the top 1% and top 0.1%, respectively) as outliers or “candidate” targets of natural selection. Depending on factors including recombination and hitchhiking, some genomic windows with extreme ΔAF that neighbored each other may have been caused by selection on the same locus or gene. Based on this, and given our aim to gain a more conservative estimate of the number of candidate loci, we merged outlier windows within 20 kb of each other and referred to them together with outlier windows as “genomic outlier regions.” Genes neighboring outlier regions within 20 kb were considered possible targets of natural selection and are reported.

To evaluate the ΔAF method we used, we also conducted genomic scans on each of the four lineages using PCadapt (Luu et al., 2016). PCadapt was run with default parameters for pool datasets except that the minimum minor allele frequency was set to 0. For consistency, PCadapt was run using the same genomic windows as evaluated in our ΔAF analysis.

### 2.4 Population genetics

We used three population genetic statistics (*D*_XY_, Tajima's *D*, and π) to analyze our pool‐seq data with two main goals in mind. First, we tested whether patterns of *D*_XY_ and Tajima's *D* in nonoutlier (neutral) and outlier regions identified at the 99.9th percentile cutoff level showed signatures of natural selection. For these comparisons, Tajima's *D* was examined separately in each of the 16 populations studied, while *D*_XY_ was averaged across all anadromous‐landlocked population pairs per lineage. Second, previous research has shown that mutations maintained by natural selection sometimes segregate in large haplotypes (Martinez Barrio et al., 2016; Nelson & Cresko, 2018; Schluter & Conte, 2009). To test whether our data fit this expectation, we estimated π for each life history type within each lineage (see Supplemental Methods).

### 2.5 Parallelism within and among lineages

We studied parallelism within the only two lineages in our study that have multiple freshwater populations, *A*.* alosa* and *A*.* f*. *lacustris* (Figure 1). To measure parallelism within them, we calculated the percentage of outlier regions shared by each pair and the set of three freshwater populations within each of them. This required us to identify outlier regions in each freshwater population of *A*.* alosa* and *A*.* f*. *lacustris*, which we accomplished using the same ΔAF methods as described above (99th percentile). Similarly, pairwise comparisons of outlier regions in populations from different lineages were conducted. We then estimated the amount of polymorphism shared by each pair of lineages. For this analysis, and throughout the text, a polymorphic site refers to any SNP we found using our methods above that was variable, or fixed for an alternative allele, in at least one of the populations (anadromous or freshwater) studied in a given lineage. Since the total number of SNP loci per lineage varied, we calculated the average of the percentage of SNPs shared in each lineage of the pair being examined. To help understand patterns of parallelism, we then compared the percentage of outlier regions shared by populations to the percentage of polymorphism common between their respective lineages.

Lastly, to help visualize patterns of genetic convergence across lineages, we generated heat maps of ΔAF for all outlier windows. One heat map was made per lineage. Specifically, for each ΔAF window in the 99.9th percentile of the target lineage, the average ΔAF for all SNPs in the same genomic window in each of the other three lineages was calculated. If the corresponding genomic window had fewer than two SNPs, it was excluded from the analysis.

### 2.6 Patterns of genetic convergence in ATPase‐α1

To explore patterns of parallel genetic evolution in these lineages in greater detail, we focused our investigation on ATPase genes that are known to be under selection in fish species (Wong et al., 2016), including alosines (Leguen et al., 2007; Velotta et al., 2017), and were outliers in our analysis. We began by using phylogenetic analysis to establish the orthology relationships of ATPase‐α1 identified in our *A*.* alosa* genome assembly. Studying comprehensive sets of gene copies from both closely related and moderately divergent species relative to the in‐group can increase accuracy when using phylogenetic methods to infer orthology (Gabaldón, 2008). Thus, most of the sequences used for the phylogeny were coding region DNA from two species for which highly complete genomic resources exist, the zebrafish (*Danio rerio*) and a clupeid (like *Alosa*), Atlantic herring (*Clupea harengus*). Sequences of alewife (*A*.* pseudoharengus*) and several outgroups were also included (see Table S2 for the sources of all sequences). These sequences were aligned using CLUSTAL X (Larkin et al., 2007) using default parameters, and phylogenetic analysis was conducted using the maximum‐likelihood (ML) method with RAxML software version 7.2.7 (Stamatakis et al., 2012). In each analysis, RAXML was set to choose the best‐fit models of nucleotide or protein substitution. The ML phylogenetic tree was reconstructed from 1000 RAXML runs, followed by 1000 bootstrap replicates.

To investigate the potential importance of three nonsynonymous substitutions in *ATPase*‐*α1*.*1b* that were found to have significant ΔAF between anadromous and freshwater *A*.* alosa* and *A*.* f*. *lacustris*, we used protein modeling and phylogenetic methods. Following Dalziel et al. (2014), the protein model used, PDB 2ZXE, was the crystal structure of dogfish (*Squalus acanthias*) (Linnaeus, 1758), Na^+^, K^+^ ATPase bound to K^+^ and MgF4^_^_2_ (Shinoda et al., 2009). The model was visualized using CCP4MG v.2.10.10 (http://www.ccp4.ac.uk). To measure how phylogenetically conserved each amino acid site is across fish species, we aligned the protein sequences for all ATPase‐α1 found in the *A*.* alosa* genome annotation and several other fish highly divergent species listed in Table S3. Finally, to help understand the significance of the ΔAF estimate for the nonsynonymous mutation in *ATPase*‐*α1*.*1b* shared by *A*.* alosa* and *A*.* f*. *lacustris* (67,614/968), we counted the number of cases where any nonsynonymous mutation shared by *A*.* alosa* and *A*.* f*. *lacustris* was also in the top 95th percentile of ΔAF (per site) between anadromous and freshwater populations in each lineage.

## 3 RESULTS

### 3.1 Genome assembly and annotation

To allow us to conduct genome‐wide scans for candidate loci in freshwater shad, we generated a draft genome of *A*.* alosa* using short‐read and mate‐pair DNA data from a single, wild‐caught individual using the ALLPATHS‐LG‐52488 assembler. The size of the *A*.* alosa* genome was estimated to be 889 Mb using the k‐mer method (Table 1). The resulting genome assembly, as measured by the total length of all scaffolds >1000 bp, was 876 Mb, indicating that it represents 98% of the entire genome. However, only 64% of the genome assembly was comprised of sequence data, with gaps making up the remaining 36%. The contig N50 of the assembled genome was short (9.9 kb). Given that 36% of the genome was estimated to be repetitive sequences (Table 1), and the limits of short‐read sequence data for resolving repeat regions (Butler et al., 2008), the short contig N50 was likely due to repetitive sequences being well distributed throughout the shad genome. Scaffolding resulted in an assembly with an N50 of 2.03 Mb, indicating that the mate‐pair data were informative for bridging contigs. Our annotation of the *A*.* alosa* genome assembly yielded 22,993 gene models. The BUSCO analysis of our annotation indicated that out of 4584 gene models tested, 71.5% were present and complete (66.0% single copy and 5.5% duplicated) and 11.3% were present and incomplete, leaving 17.2% missing. While the assembly was sufficiently complete to identify hundreds of candidate regions in each lineage, a significant proportion of loci involved in adaption may have been missed.

**TABLE 1:** Statistics from the ALLPATHS genome assembly for *A*.* alosa*

Estimated genome size (bp)	889,386,068
Assembly size (bp)	876,687,730
Scaffold N50 (bp)	2,036,090
Contig N50 (bp)	9,900
Total contig length (bp)	621,125,889
No. of scaffolds >1000 bp	15,886
Repeat content	36%
% GC content	43.4%
Heterozygosity	1/230
Protein‐coding gene count	32,983

### 3.2 Whole‐genome sequencing and genome scans for candidate loci

A crucial assumption of our tests for parallelism is that the lineages and populations studied have independently adapted to freshwater. Our phylogenetic analysis (Figure 1b) and previous work (Faria et al., 2012) make it clear that the lineages studied evolved independently. The freshwater populations of *A*.* alosa* we examined were all founded after being trapped behind dams built in unconnected drainages during the past century (Aprahamian et al., 2003), and their independence has been confirmed by genetic analysis here, and more comprehensively in Faria et al. (2012) and Sabatino et al. (2022). The evolutionary history of the Italian lake populations examined, however, is less clear. Our analysis is consistent with previous work showing they all were founded by anadromous individuals in the Po River and therefore are closely related (Faria et al., 2012; Sabatino et al., 2022). The geologic record suggests that each of the lakes that these freshwater *A*.* f*. *lacustris* now inhabit, and the rivers into which they flow, was not directly connected during or after the Pleistocene (Castaldini et al., 2019). However, given their close relationship and that post‐colonization gene flow among them was possible, these freshwater populations may not be independent. Accordingly, the statistical approach used to conduct genome scans for outlier loci does not assume independence among freshwater populations within lineages (see below).

To study parallelism, we conducted whole‐genome resequencing of pools of DNA (“pool‐seq”) from 16 populations of shad representing four different lineages of *Alosa*. This included generating pool‐seq data for at least two anadromous, and from one, or three, freshwater populations, per lineage. The pool‐seq data were then used to estimate allele frequencies for SNP loci that were then used for population genetic analysis. The highest number of SNPs was found in the *A*.* f*. *killarnensis* lineage (~1.9 M), approximately two to three times higher than the other three lineages (*A*.* alosa* ~0.6 M; *A*.* f*. *lacustris* ~0.8 M; *A*.* macedonica*: ~0.9 M) (Tables S1 and S4).

**FIGURE 2:**
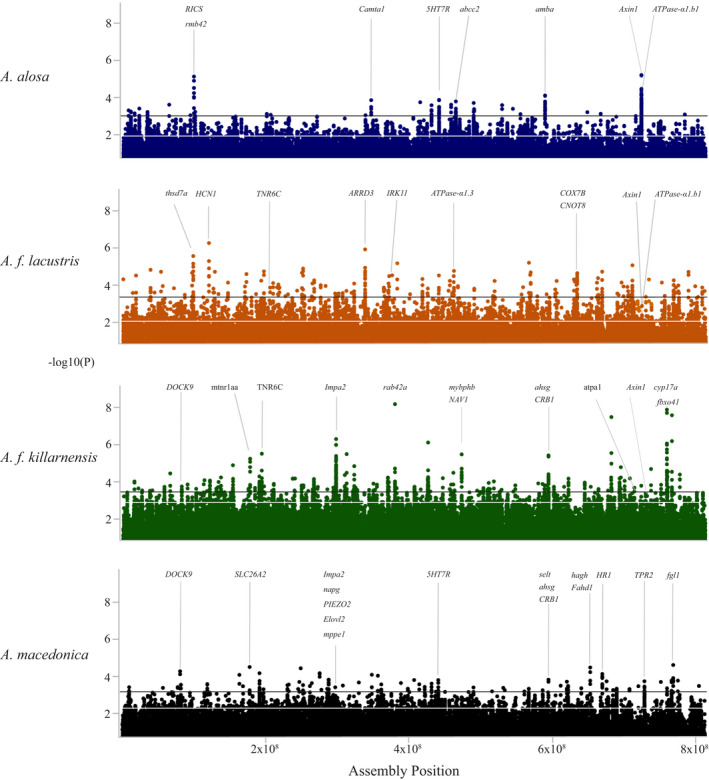
Manhattan plots showing the significance of ΔAF between anadromous and freshwater populations of the four lineages studied: *A*.* alosa*—Atlantic (navy), *A*.* f*. *killarnensis*—Ireland (green); *A*.* f*. *lacustris*—Italy (orange); and *A*.* macedonica*—Black Sea (black). On the *x*‐axis, the SNPs are ordered based on their position in each scaffold from our *A*.* alosa* genome assembly, with the largest scaffolds on the left and the smallest on the right. The *y*‐axis shows ‐log(10) *p*‐values calculated using 1000 permutations of the data per lineage. The horizontal lines indicate the 99.9th (black) and 99th (white) percentiles

Allele frequencies for the resulting SNP loci were then used to conduct genome scans for candidate loci in each of the four lineages studied using a sliding window approach and permutation tests. The effect of natural selection on SNP allele frequencies can be highly variable even when selection is strong (Wilson et al., 2014). We conducted permutation tests to identify outliers using two different statistical thresholds (99th and 99.9th percentiles) (Figure 2; Table 2; Supplemental Text; Table S5). To try to avoid overestimating the number of candidate genomic locations identified in our genome scans, and help identify overlapping outlier regions in different lineages, outlier windows within 20 kb of each other were merged into outlier genomic regions. This resulted in a roughly twofold to a sixfold reduction in the number of genomic areas identified as candidates (Table 2).

**TABLE 2:** Numbers of outlier windows (20 consecutive SNPs) and regions (outlier windows within 20 kilobases of each other) per lineage and population(s) studied

Lineage	Population	99th percentile		99.9th percentile	
		Windows	Regions	Windows	Regions
*A*.* alosa*	All	1394	397	140	36
	Aguieira Reservoir		398		37
	Castelo de Bode Reservoir		412		57
	Alqueva Reservoir		431		31
*A*.* f. lacustris*	All	1568	554	157	85
	Lake Maggiore		609		83
	Lake Como		556		77
	Lake Garda		581		79
*A*.* f. killarnensis*	Lough Leane	2173	552	218	70
*A*.* macedonica*	Lake Volvi	1428	491	143	70

Genes in the outlier regions identified in each lineage have a variety of biological functions including osmoregulation, apoptosis, stress response, metabolism, brain and eye development, muscle growth, and immunity (Figure S1; Table S5; see Supplemental Text), some of which had previously been shown to be involved in adaptation to osmotic stress or life history changes in *Alosa* or other fish species (e.g., *ATPase*‐*α1* (Velotta et al., 2017); *IMPA2* (Gardell et al., 2013)). Candidate genes were often in locations with relatively low genetic diversity (Figure S2), further supporting the hypothesis that they are under selection.

To evaluate our ΔAF method for conducting genomic scans, we used PCadapt. The number of population clusters (k) per lineage identified using PCadapt and used for the outlier analysis was two in all cases except for *A*.* alosa* where four better fit the data (Figure S3). The percentage of outlier widows identified in our ΔAF analysis that overlapped with those found using PCadapt ranged from 75% to 100% (99.9th percentile in the ΔAF analysis) and from 57% to 89% (99th percentile) (Table S6; Figure S4), indicating a generally high level of congruence between the two methods. Differences between them were likely attributable to how population structure impacts the results of each method. For example, in the PCadapt analysis of the *A*.* f*. *lacustris* lineage, one of the main drivers of differentiation in the first component is the genetic distance between the two anadromous populations studied, which may have diluted the signal from contrasts between them and the freshwater populations examined. While our ΔAF analysis is also impacted by neutral evolutionary history, it was designed to minimize its effect and to identify outliers specifically associated with adapting to a completely freshwater life cycle. Additionally, based on the several‐fold greater number of outlier windows identified using PCadapt (Table S6), our ΔAF method is the more conservative of the two. We therefore note outlier regions that were identified by both methods (Table S5) but conducted our full analysis of parallelism using the results of our ΔAF outlier analysis.

### 3.3 Population genetics

We studied each lineage using *D*_XY_, Tajima's *D*, and π. Divergence (*D*_XY_) between anadromous and freshwater populations was consistently higher in outlier regions compared with nonoutlier ones in all four lineages (Figure S6a). Mean Tajima's *D* was below zero in all 16 populations examined (Figure S7), suggesting they may have experienced range expansions, as was found for several of the populations studied here using mtDNA data (Faria et al., 2012). Bias in our estimation of rare alleles, which can occur using pool‐seq data (Nielsen et al., 2012), may also have contributed to low estimates of Tajima's *D*. Tajima's *D* were significantly lower in outlier regions in *A*.* f*. *lacustris*, *A*.* f*. *killarnensis*, and *A*.* macedonica*, but not in *A*.* alosa* except for one of the anadromous populations examined, where the opposite trend was found. The different pattern in Tajima's *D* found for *A*.* alosa* than the other three lineages could be due to the young age of its freshwater populations (tens of generations), as they are unlikely to be at genetic equilibrium. Nonetheless, the observed patterns of Tajima's *D* in all three naturally occurring lineages, and those for *D*_XY_ for all four of them, suggest that the outlier regions examined are targets of natural selection.

Differences in π within and outside of outlier regions were detected in five of the eight comparisons we conducted (Figure S7b). In *A*.* alosa* and *A*.* f*. *lacustris*, outlier regions in both anadromous and freshwater populations showed a significant excess in diversity when compared to neutral ones, suggesting they may be enriched for older haplotypes that contain multiple mutations that are under selection. However, the opposite pattern was found for freshwater *A*.* macedonica*. No significant differences were detected in the remaining three comparisons conducted.

### 3.4 Genetic parallelism within and across lineages

**FIGURE 3:**
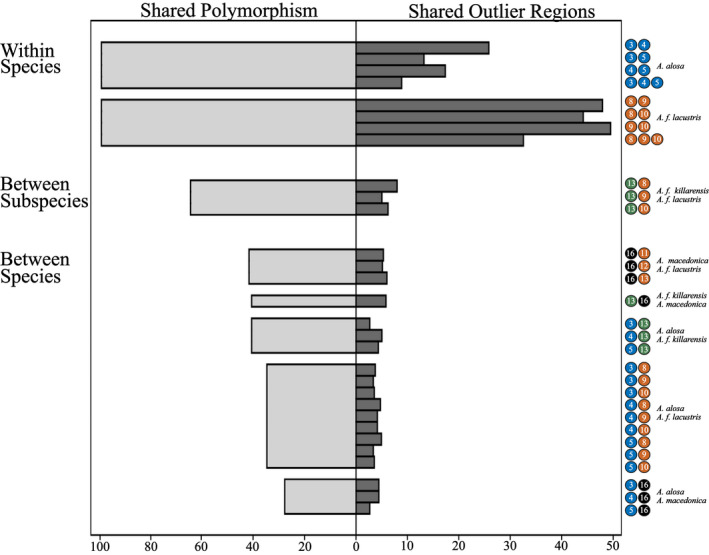
Percentage of polymorphism shared compared with the percentage of outlier regions (99th percentile) in common for pairs and sets of freshwater populations studied. The colored circles with numbers refer to populations as shown in Figure 1

Parallelism was measured within *A*.* alosa* and *A*.* f*. *lacustris*, and among all four of the lineages examined. The percentage of shared outlier regions between pairs of populations within *A*.* alosa* was generally lower (an average of 18%) than *A*.* f*. *lacustris* (48%), as was it across all three populations in each lineage (Figure 3). However, pairwise estimates within both species were generally several‐fold higher than between freshwater populations of any of the four lineages studied. The geographic/genetic distance between the anadromous and freshwater populations studied in each lineage makes it possible that some of the outlier regions identified were caused by selection due to factors other than adapting to freshwater (e.g., temperature). Additionally, predicting how neutral evolutionary history such as secondary contact will affect the number of shared outlier regions among populations and lineages was not assessed. While these issues do limit our study, congruence between our genome‐wide analyses of parallelism and that of *ATPase*‐*α1*.*1b* (see below) provides support for our overall conclusions regarding this topic.

Parallelism among lineages was then compared with the amount of shared polymorphism between them. The percentage of polymorphism shared was highest for the two subspecies examined, *A*.* f*. *lacustris* and *A*.* f*. *killarnensis* (64%), and lowest between *A*.* alosa* and *A*.* macedonica* (25%) (Figure 3). Despite the subspecies examined sharing from 30% to 50% more polymorphism than any of the other lineages, parallelism was slightly higher for the two subspecies than in any of the cross‐species comparisons.

In addition, only a fraction of the hundreds of thousands to millions of SNPs found per lineage were shared by more than two lineages. For example, around 70 thousand shared SNP loci among the three naturally landlocked groups (*A*.* f*. *lacustris*, *A*.* f*. *killarnensis*, and *A*.* macedonica*), and just several thousand SNPs were shared among all four lineages of freshwater shad (see Table S7). In several cases, the same genomic region was found to be an outlier in three of the lineages (Figures S1 and S2; Table S6). No genomic region was an outlier in all four of the lineages studied.

### 3.5 Evolutionary analysis of ATPase‐α1.1b

To help understand the genome‐wide patterns of parallel genetic evolution identified here, we focused on a gene that is likely to be a target of selection in the lineages we studied, *ATPase*‐*α1*.*1b*. First, phylogenetic analysis was used to infer the orthology of all ATPase‐α1 identified in our study. Statistical support for most internal nodes in the maximum‐likelihood trees generated for each gene was significant (>70% bootstrap support; Figure S7). The analysis revealed that the ATPase‐α found to be a top outlier in *A*.* f*. *lacustris* was likely *ATPase*‐*α1*.*3*. It also confirmed *ATPase*‐*α1*.*1*b, which was found to be a candidate gene in *A*.* alosa* and *A*.* f*. *lacustris*, was an ortholog of the *ATPase*‐*α1* identified as under selection in Velotta et al. (2017).

**FIGURE 4:**
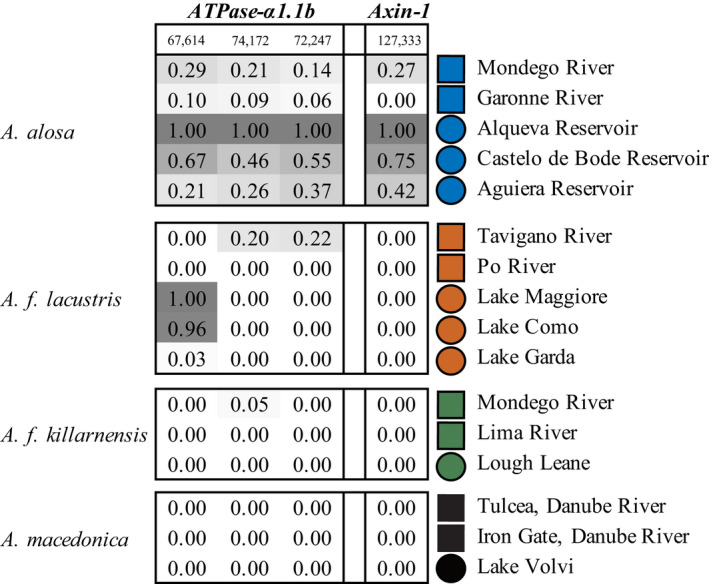
Heat map of allele frequencies for nonsynonymous mutations found in *ATPase*‐*α1*.*1b* and *Axin1*‐*like*. The number atop each column is the location of the SNP in scaffold_411 in our *A*.* alosa* genome assembly. At the end of each row is the name of the population and a symbol indicating if it is anadromous (squares) or freshwater (circles). Each cell is shaded gray based on the frequency of the “freshwater” allele

**FIGURE 5:**
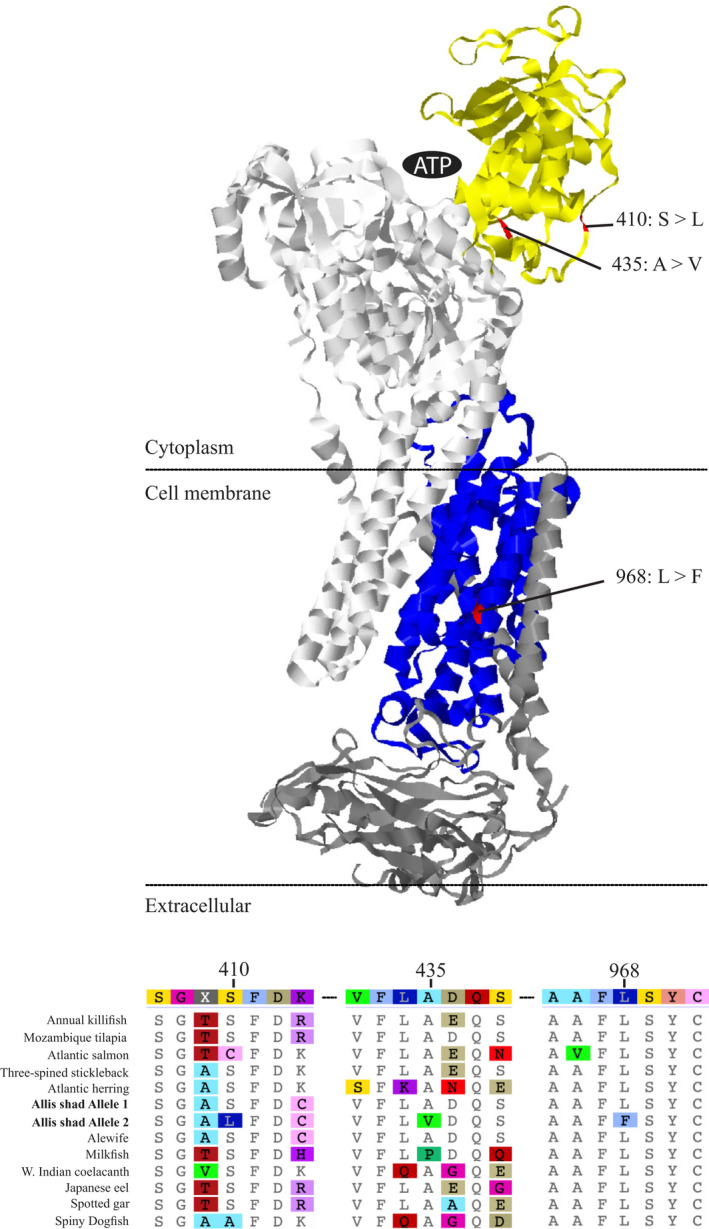
3D‐model of Na,K‐ATPase and subsections of an amino acid alignment of *ATPase*‐*α1* from several fish species. In the alignment, the alternative alleles for the three protein‐coding changes found in freshwater populations of *A*.* alosa* are shown. In the protein model, the domains where these three mutations occur are highlighted in yellow (nucleotide‐binding) and blue (transmembrane). The rest of *ATPase*‐α is colored white, while *ATPase*‐β and *ATPase*‐γ are both dark gray. The numbering of amino acid positions corresponds to that used in Shinoda et al. (2009) for the spiny dogfish

Among the mutations in *ATPase*‐*α1*.*1*b with extreme ΔAF between anadromous and freshwater populations of *A*.* alosa* and *A*.* f*. *lacustris* were three that were nonsynonymous. One of these three mutations, 67,614/968 (positions in Figures 4 and 5), was an outlier in both *A*.* alosa* and *A*.* f*. *lacustris* with ΔAF between 0.6 and 1.0 in two of three freshwater populations in each lineage, and a third exhibiting more moderate changes (~0.3). In *A*.* alosa*, two additional nonsynonymous mutations in *ATPase*‐*α1*.*1*b that also exhibited extreme ΔAF (74,172/435 and 74,247/410) were found. To further understand the statistical significance of the ΔAF estimate for the nonsynonymous mutation in *ATPase*‐*α1*.*1*b shared by *A*.* alosa* and *A*.* f*. *lacustris*, 67,614/968, we examined the genome‐wide distribution of ΔAF for all nonsynonymous mutations shared by the two lineages. Among all nonsynonymous mutations shared by the two lineages, 67,614/968 in *ATPase*‐*α1*.*1*b had the highest combined ΔAF. It was also the only one with ΔAF in the top 95^th^ percentile of all ΔAF for all nonsynonymous mutations in each *A*.* alosa* and *A*.* f*. *lacustris*. All three nonsynonymous changes in *ATPase*‐*α1*.*1*b had similar allele frequencies in all populations studied, suggesting they may be part of the same haplotype.

The functional consequences of these three nonsynonymous mutations were explored using protein modeling and phylogenetic analysis. Based on our amino acid alignment of *ATPase*‐*α1* from a phylogenetically diverse set of fish species, each of the three positions that had nonsynonymous mutations in *ATPase*‐*α1*.*1*b is moderately conserved loci (Figure 5). The amino acid change at position 968 from leucine (L) to phenylalanine (F) is nearby to an inserted or retained amino acid (position 967). This insertion is absent in *ATPase*‐*α1* of most fish species but found in a minority of copies of the gene present in clupeids and other euryhaline fish such as some salmonids and killifish. Protein modeling of the three nonsynonymous mutations indicated that the two nonsynonymous mutations that were outliers in just *A*.* alosa* (positions 410 and 435 in our amino acid alignment) are part of the same loop within the nucleotide‐binding domain, suggesting they may work in concert to alter how ATP binds to the protein (Figure 5). The amino acid change shared by *A*.* alosa* and *A*.* f*. *lacustris* (position 968) was found to be in the cell membrane between the cytoplasm and extracellular regions where it may affect ion transport.

## 4 DISCUSSION

Here, we studied the genomics of four lineages of anadromous Eurasian shad that have evolved freshwater life histories. Our results show that adaptation to freshwater in these alosines involved a combination of parallel and independent genetic changes. Parallelism varied extensively at each of the two phylogenetic scales investigated but was generally greater within lineages than among them.

### 4.1 Parallelism within lineages

Multiple freshwater populations exist for two of the four *Alosa* lineages we studied (*A*.* alosa* and *A*.* f*.* lacustris*), making it possible to investigate parallelism within each of these. The freshwater *A*.* alosa* studied here have only been diverging for tens of generations. Our evidence for parallelism in *A*.* alosa* (Figure 3, Supplemental Text, Figure S2) adds to a growing body of literature, showing that adaptation can rapidly occur (Bell et al., 2004; Hairston et al., 2005). We also found support for parallelism among freshwater populations of *A*.* f*. *lacustris*. Within *A*.* alosa* and *A*.* f*. *lacustris*, genomic outlier regions common to all freshwater populations studied were identified (i.e., “complete parallelism”). However, in *A*.* alosa*, complete parallelism was less common than in *A*.* f*. *lacustris*.

Greater parallelism, overall, within *A*.* f*. *lacustris* than *A*.* alosa* is consistent with freshwater *A*.* f*. *lacustris*: (1) having probably been founded by anadromous shad from the same drainage (Sabatino et al., 2022), (2) potentially diverging with gene flow and not being completely independent, and (3) inhabiting lakes with similar environments (e.g., temperature, water chemistry, and predators) (Salmaso et al., 2012; Salmaso & Mosello, 2010). In contrast, the freshwater *A*.* alosa* populations studied were each founded by different anadromous ones just tens of generations ago and without gene flow. This scenario resulted in parallelism within *A*.* alosa* that was less than found among the four lineages examined but lower than in *A*.* f*. *lacustris*. Allele frequencies can take hundreds of generations to reach equilibrium in response to selection (Star & Spencer, 2013). It is therefore likely that lower parallelism in *A*.* alosa* is due at least in part to divergence time. Additionally, the two freshwater populations with the highest number of shared outlier regions were founded by the two (of the three involved) most closely related anadromous ones (Sabatino et al., 2022). This suggests that within this lineage, in the absence of gene flow, similarity in the gene pools of the founding populations can increase the likelihood of parallelism. However, these freshwater populations are just decades old, and allele frequencies for traits under selection due to their recent ecological expansion may still be changing. Future studies should examine whether and how parallelism within *A*.* alosa* shifts over time to further test these hypotheses.

### 4.2 Parallelism among lineages

Our results indicate that parallelism among the lineages examined is not widespread. No evidence for parallelism between lineages was found using our most conservative statistical cutoff for identifying outlier genomic regions (99.9th percentile). At the 99th percentile level, pairs of populations from different lineages shared <5% (hundreds) of their outlier regions (Figure 3). Fewer regions (from one to six) were shared by sets of three lineages (Table S6). Together, these results show that adaptation to freshwater is often independent across these lineages. Of the millions of SNPs that we identified across the four lineages here, the number common to all was in the thousands. For each of the three lineages, this number was in the tens of thousands (Table S7). This makes it plausible that the (low) level of shared polymorphism across the lineages examined is a limiting factor to parallelism.

If shared polymorphism promotes parallelism across these lineages, it should be highest for *A*.* f*. *lacustris* and *A*.* f*. *killarnensis* since they are the most closely related of the four examined. While this was found to be the case (Figure 3), the proportion of overlapping regions shared between populations of each subspecies was only marginally higher than between those of different species. While we cannot rule out bias in our results due to the methods used to identify outlier regions and measure shared polymorphism (e.g., coverage, sampling), and the low number of lineages examined, our results suggest that shared polymorphism is not a strong driver of parallelism across these four *Alosa* lineages. This hypothesis was supported by our focused analysis of parallelism involving *ATPase*‐*α1*.*1*b.

### 4.3 Parallelism for ATPase‐α1

Several lines of evidence indicated that *ATPase*‐*α1*.*1*b is under selection in *A*.* alosa* and *A*.* f*. *lacustris*. Among the nucleotide sites in *ATPase*‐*α1*.*1*b that exhibited extraordinary ΔAF in *A*.* alosa* were three nonsynonymous mutations, one of which also sits at the tip of sharply peaked outlier regions in two of the three *A*.* f*. *lacustris* populations (Figure S2). This was also the only one of the three nonsynonymous mutations found in outlier regions of both *A*.* alosa* and *A*.* f*. *lacustris*. Using protein modeling, this same mutation was predicted to change an amino acid that affects a transmembrane domain of Na+/K+ATPase (Figure 5) and thus may play a functional role in ion pumping. Lastly, *ATPase*‐*α1*.*1*b was shown to be orthologous to an *ATPase*‐*α1* that is differentially expressed in freshwater versus anadromous *A*.* pseudoharengus* in common garden experiments involving osmotic stress (Velotta et al., 2017). Together, these results suggest that *ATPase*‐*α1*.*1*b is under selection in *Alosa* species and that in *A*.* alosa* and *A*.* f*. *lacustris*, it involves protein‐coding changes. Ion transport involving ATPases is an active process whose efficiency can be affected by temperature (Galarza‐Munoz et al., 2011). Mismatches between *ATPase*‐*α1*, environmental conditions, and the migratory behaviors of shad may thus significantly decrease the efficiency, or increase the energetic costs, of biological processes such as osmoregulation, resulting in them being under natural selection in these lineages.

However, *ATPase*‐α1 is expressed in a variety of tissues and plays roles in other biological processes that likely affect fitness in shad. For example, *ATPase*‐α1 is involved in brain development in zebrafish (Doğanlı et al., 2013; Rajarao et al., 2001). Functional assays to determine the fitness effects of the nonsynonymous mutations found in *ATPase*‐*α1*.*1*b may help to characterize the selective forces acting on them in shad.

Despite the only two subspecies we examined, *A*.* f*. *lacustris* and *A*.* f*. *killarnensis*, sharing the most polymorphism of any two lineages within our study, *ATPase*‐*α1*.*1b* was not an outlier in *A*.* f*. *killarnensis*. Moreover, the nonsynonymous *ATPase*‐*α1*.*1b* alleles found were not found in any of the populations (anadromous or freshwater) studied in the *A*.* f*. *killarnensis* lineage. This provides a clear example of how the genetic distance and overall shared polymorphism is not a good predictor of parallelism within these lineages.

The observed patterns of genetic evolution in *ATPase*‐*α1*.*1b* across populations within *A*.* alosa* and *A*.* f*. *lacustris* may provide some clues as to what may influence parallelism in these lineages. Although the genomic regions that contained *ATPase*‐*α1*.*1*b in *A*.* alosa* and *A*.* f*. *lacustris* exhibited some of the most extreme ΔAF observed, such changes were not consistently observed in all freshwater populations of each lineage (Figure 4). The putatively freshwater‐adapted *ATPase*‐*α1*.*1*b allele for locus 67614/968 (Figure 4) was present in all six of these freshwater populations and so suggests that lack of shared polymorphism is not the underlying cause of incomplete parallelism in these cases. Partial parallelism here may have been driven by drift outweighing the effect of selection in some populations. However, a lack of adaptive change due to the effects of drift and/or weak selection would have had to occur independently in each lineage, making it a less parsimonious explanation.

One alternative hypothesis is that antagonistic genetic interactions have shaped the observed patterns in allele frequencies for *ATPase*‐*α1*.*1*b and/or genes linked to it. Fitness tradeoffs and trait associations involving Na^+^ K^+^‐ATPase and osmoregulation and migration have been identified in *Alosa* (Velotta et al., 2015; Zydlewski & McCormick, 1997) including *A*.* alosa* (Leguen et al., 2007), and other anadromous/freshwater species such as salmon (Hecht et al., 2013) and stickleback (Hohenlohe et al., 2010). A common characteristic of loci experiencing antagonistic pleiotropy is that the polymorphisms involved are under balancing selection polymorphisms in some ecological settings (Connallon & Clark, 2012). Intermediate allele frequencies for SNPs in and around *ATPase*‐*α1*.*1*b were observed for both anadromous populations studied in the *A*.* alosa* lineages, and one in the *A*.* f*. *lacustris*, exhibited, providing support for this hypothesis.

However, the high ΔAF for SNPs in other genes adjacent to *ATPase*‐*α1*.*1*b such as Mk2 and *Axin1*‐*like* (Figure 4) indicate that the evolution of this genomic region may be more complicated. The allele frequencies for most SNPs in this region, including the nonsynonymous ones *ATPase*‐*α1*.*1*b and *Axin1*‐*like*, had similar allele frequencies in different populations, suggesting linkage disequilibrium among them and the potential existence of a large, multi‐gene haplotype that is under selection. Like *ATPase*‐*α1*.*1*b, *Axin1* is involved in brain development (Carl et al., 2007), making it plausible they are under similar selection pressures. If so, the ultimate effects of selection in each lineage may be difficult to predict (Hill & Robertson, 1966).

Indeed, genetic divergence (*D*_XY_) was consistently higher within outlier regions (including *ATPase*‐*α1*.*1*b) identified in *A*.* alosa* and *A*.* f*. *lacustris* (Figures S2 and S6). This suggests that they are comprised of ancient haplotypes, as was found for outlier regions in herring and stickleback (Martinez Barrio et al., 2016; Nelson & Cresko, 2018). In the case of *A*.* alosa*, this is likely the case given the populations were founded just tens of generations ago, and little time has passed for new mutations to occur. In herring, outlier regions also had elevated diversity (π), which was interpreted as them containing multiple causal mutations (Martinez Barrio et al., 2016). This same pattern was found for outlier regions in freshwater *A*.* alosa* and *A*.* f*. *lacustris* (Figure S6b) suggesting they may contain haplotypes with multiple loci under natural selection. The existence of several nonsynonymous mutations with extreme ΔAF in the outlier region containing *ATPase*‐*α1*.*1*b and *Axin1* supports this hypothesis.

### 4.4 Parallelism in Eurasian shad and other species

Parallelism within *A*.* alosa* and *A*.* f*. *lacustris* found here appears lower than in stickleback and some strictly marine species but similar to that in other anadromous and freshwater organisms. For example, a high degree of parallelism exists for multiple loci across replicate populations of freshwater‐adapted stickleback (Cresko et al., 2004; Hohenlohe et al., 2010; Magalhaes et al., 2021). In the marine snail, *Littorina saxatilis*, around 15% of outlier windows were shared among five or more pairs of populations of ecotypes (Morales et al., 2019). Also, in codfish and herring, genes involved in thermal adaptation and reproduction timing, respectively, showed consistent signs of parallel evolution (Bradbury et al., 2010; Lamichhaney et al., 2017). By contrast, like in *A*.* alosa* and *A*.* f*. *lacustris*, complete parallelism in alewife (Velotta et al., 2017), salmonids (Hecht et al., 2013; Jeffery et al., 2017), whitefish (Renaut et al., 2012), and cichlids (Elmer et al., 2014; Meier et al., 2018) was rare. One set of factors that may help explain these broadscale differences is that effective population size and genetic structure are generally higher, and gene flow is lower, in anadromous species such as shad, and those in freshwater, compared with stickleback, whitefish, herring, and snails (Waples, 1998). Indeed, at higher geographic scales (Fang et al., 2020, 2021; Magalhaes et al., 2021; Roberts Kingman et al., 2021), and when *N*_E_ or genetic diversity is limited due to historical factors (Dahms et al., 2022), parallelism in stickleback is more moderate than at the regional or local scales.

However, the observed disconnect between parallelism and shared polymorphism in the alosines studied here also highlights how the situation may often be more complicated. One possible explanation is secondary contact. Introgressed alleles are often involved in adaptation (Hedrick, 2013), and hybridization among contemporary populations *A*.* alosa* and *A*.* fallax* has resulted in gene flow among them (Aprahamian et al., 2003; Faria et al., 2012; Taillebois et al., 2019). If introgression from hybridization occurred during the divergence of each lineage, our results indicate it was insufficient to maintain a level of shared polymorphism that strongly promotes parallelism. However, introgressed alleles under balancing selection will often have longer coalescence times than neutral ones (Charlesworth et al., 1997). Introgression may have thus significantly altered the geographic distribution of fitness‐related alleles available for adaptation (and parallelism) in these lineages. If introgressed alleles were often involved in adaptation to freshwater in these lineages, then genetic distance or neutral shared polymorphism would poorly predict patterns of parallelism, as has been found.

Parallelism across lineages may have also been limited by environmental factors. Environmental similarity is a strong driver of parallelism in some species (Magalhaes et al., 2021). While anadromous individuals colonized each freshwater location studied, their life histories, phenotypes, and habitats in the Atlantic, Mediterranean, and Black Sea are far from identical. At the same time, while the freshwater shad we studied tend to phenotypically converge, they differ in some traits (e.g., size and parity) and inhabit contrasting freshwater environments ranging from the alkaline lakes in the foothills of the Italian Alps to recently formed reservoirs in Portugal. Accordingly, anadromous individuals may have required lineage‐specific, or freshwater habitat‐specific, genetic changes to evolve a freshwater life history. It is also noteworthy that the freshwater *A*.* alosa* studied adapted to a freshwater history in a single generation, serving as a remarkable example of the well‐known physiological flexibility and plasticity of the genus concerning their life histories and osmoregulation (Aprahamian et al., 2003). This plasticity may have resulted in weaker selection for traits that evolved in response to ecological changes experienced by all freshwater populations in each lineage, such as osmoregulation in freshwater as adults. If so, allele frequency changes resulting from lineage‐ or drainage‐specific adaptations due to comparatively intense selective pressures may have often overshadowed them in our analysis.

## AUTHOR CONTRIBUTIONS

**Stephen J. Sabatino:** Conceptualization (lead); data curation (lead); formal analysis (lead); funding acquisition (lead); investigation (lead); methodology (lead); writing – original draft (lead); writing – review and editing (lead). **Paulo Pereira:** Formal analysis (equal); investigation (supporting); writing – original draft (supporting); writing – review and editing (equal). **Miguel Carneiro:** Investigation (supporting); methodology (supporting). **Jolita Dilytė:** Methodology (supporting). **John Patrick Archer:** Data curation (supporting); formal analysis (supporting). **Antonio Munoz:** Formal analysis (supporting). **Francesco Nonnis‐Marzano:** Resources (supporting). **Antonio Murias:** Data curation (equal).

## CONFLICT OF INTEREST

All authors declared that there is no conflict of interest.

## Supporting information

Appendix S1

## Data Availability

The DNA sequence data for the genome assembly and pooled sequencing in this article are available on GenBank: BioProject PRJNA649106 ‐ Genome Assembly (Biosample: SAMN27776347; overlapping library: SRR18919708; mate‐pairs: SRR18964345‐SRR18964349); Pooled sequencing (SAMN27767898‐SAMN27767913). The genome annotation can be found at https://doi.org/10.5061/dryad.6djh9w13n
