# Supplementary material for: The genetics of adaptation in freshwater Eurasian shad (Alosa)
Source: Ecol Evol. 2022 May 24;12(5):e8908. doi: 10.1002/ece3.8908 (PMC9130566; doi:10.1002/ece3.8908)
Supplement: Supplementary file 1 — Appendix S1 [file ECE3-12-e8908-s001.pdf]

Supplemental Figure 1. Heat map of differences in allele frequencies (ΔAF) for genomic windows with extreme ΔAF values (top 0.1%) per lineage and corresponding ΔAF in each of the other three lineages studied. The ΔAF are colored so that yellow shows the lowest values and blue is highest. White blocks contained too few SNPs to estimate ΔAF and therefore were omitted. Two-letter codes at the top of each column refer to lineages as follows: "AA" – *A. alosa*, "AFL" – *A. f. lacustris*, "AFK" – *A. f. killarnensis* and "AM" – *A. macedonica*. The closest gene to each outlier window, within a maximum of 20 kilobases up or downstream away, is shown.

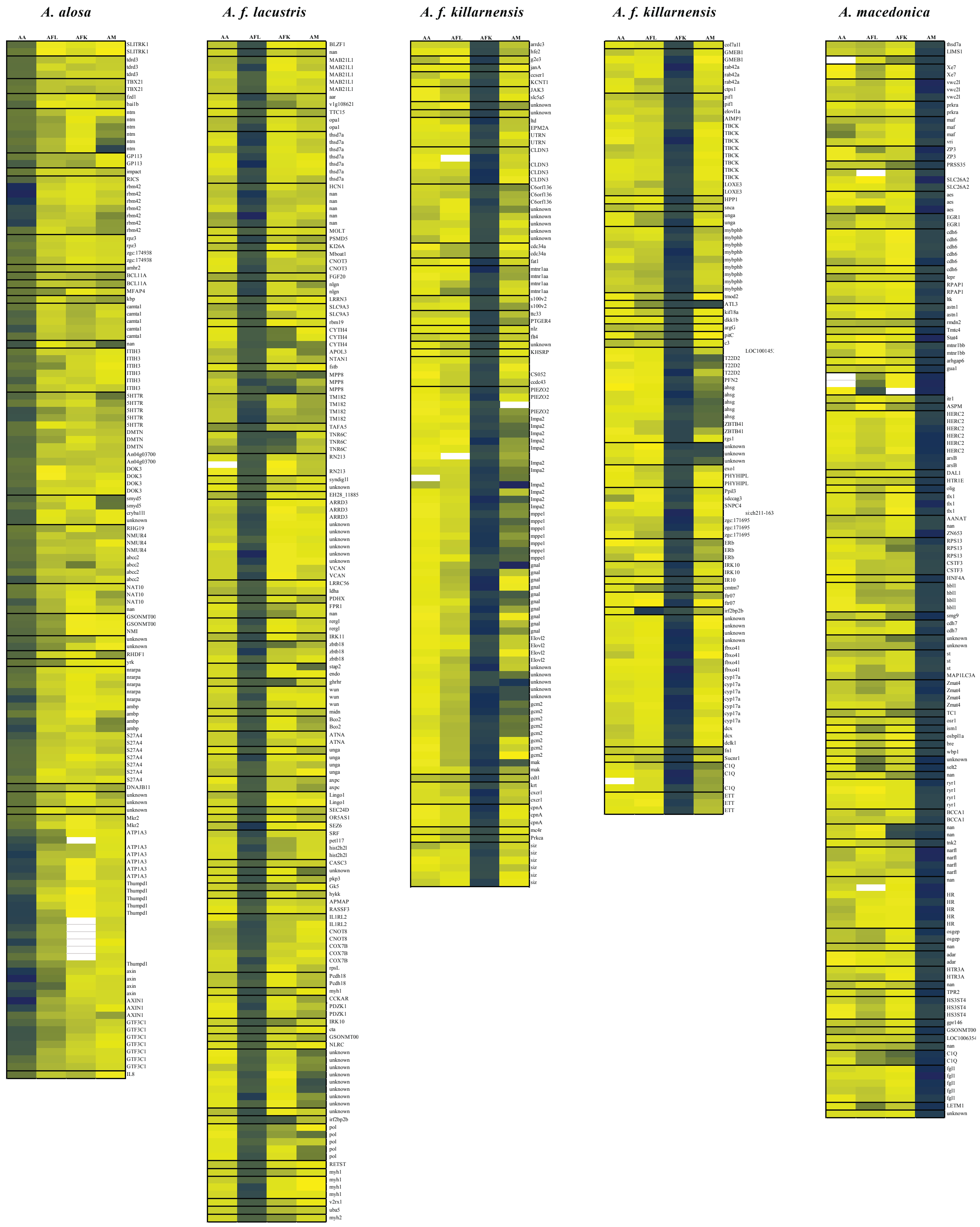

Supplemental Figure 2. Plots of differences in allele frequencies (ΔAF) for genomic windows per population for all four lineages studied for several candidate genes identified in this study. The plots are colored as follows: *A. alosa* – Atlantic (Navy), *A. fallax* – Ireland (Green), *A. fallax* – Italy (Orange), and *A. macedonica* – Black Sea (Black). Triangles mark the location of each gene or pair of genes named at the top of each plot. The plot at the bottom of each row shows the average genetic diversity (π) in 20 kilobase windows with a step size of ten kilobases.

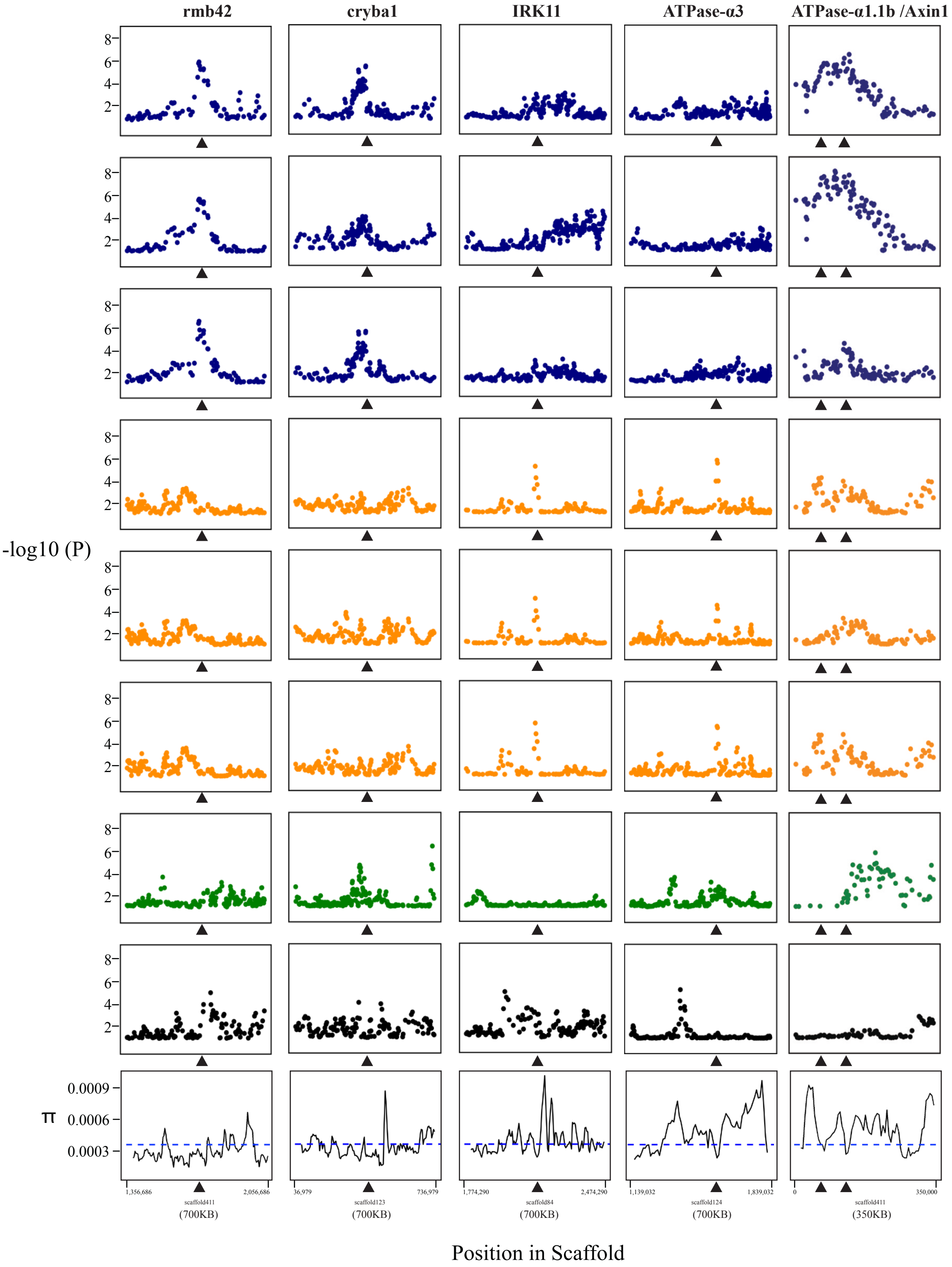

Supplemental Figure 3. Principle Components Analysis from PCadapt for two of the lineages studied. The numbered squares (anadromous) and circles (freshwater) are populations as shown in Figure 1.

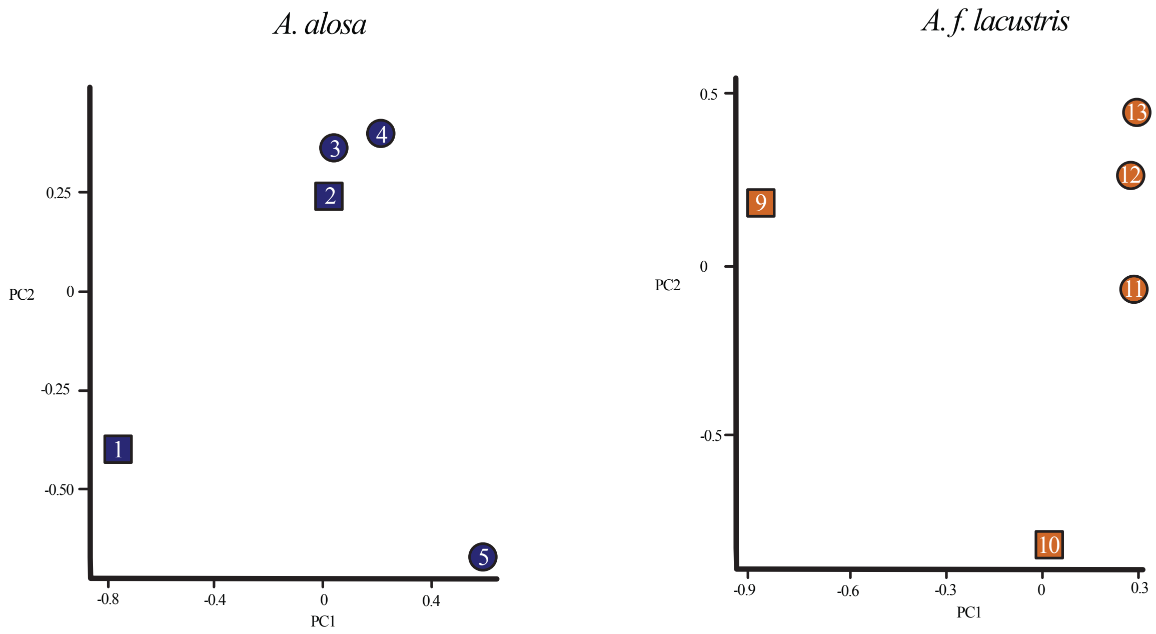

Supplemental Figure 4. Manhattan plot of p-values for PCadapt (top of each panel) and allele frequency differences (ΔAF) per genomic window (bottom) between anadromous and freshwater populations of the four lineages studied: *A. alosa* – Atlantic (Navy), and *A. f. killarnensis* – Ireland (Green), *A. f. lacustris* – Italy (Orange), *A. macedonica* – Black Sea (Black). On the X-axis, the SNPs are ordered based on their position in each scaffold from our *A. alosa* genome assembly, with the largest scaffolds on the left and the smallest on the right.

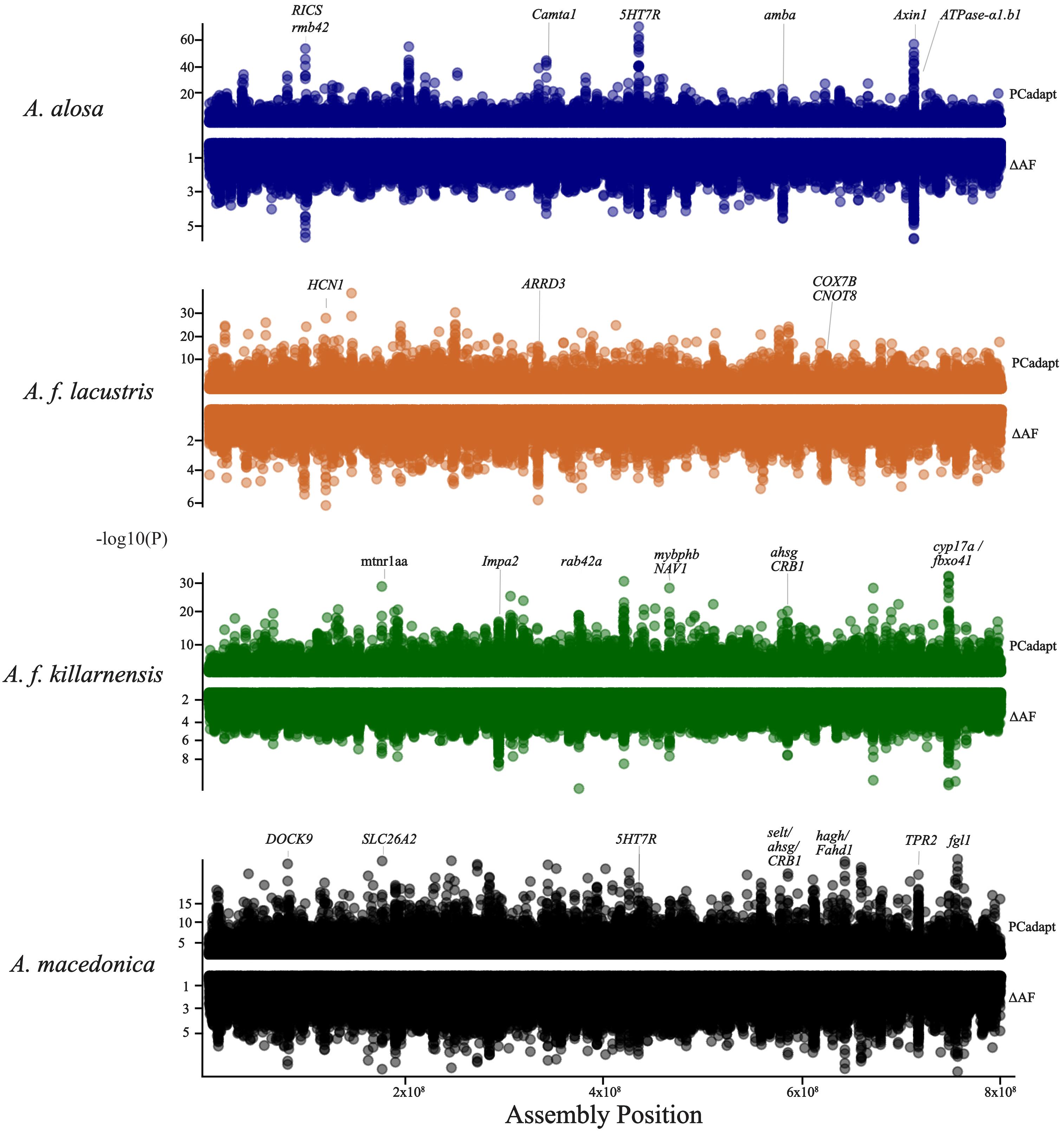

Supplemental Figure 5. Phylogenetic tree of the ATPase genes for several fish species including *A. alosa*. Genes found in our *A. alosa* assembly are shaded grey. A star is used to mark ATPase genes found to be candidates in our genome scan analysis.

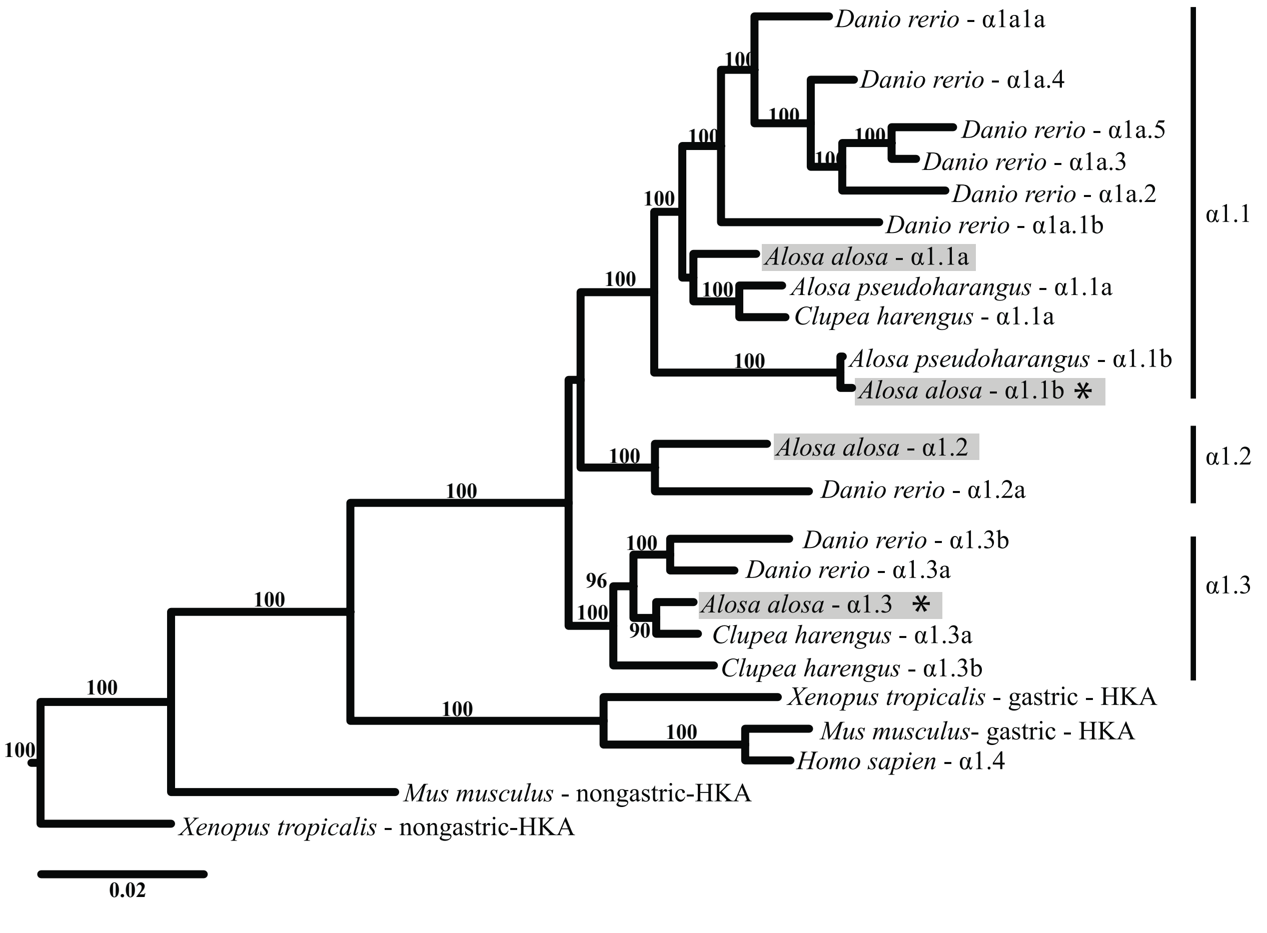

Supplemental Figure 6a and 6b. Comparisons of nucleotide diversity, π, (a) and absolute genetic divergence, D_{XY}, (b) within (+) and outside (-) of outlier regions. The analysis of π was done separately for anadromous and freshwater populations within each of the four lineages studied. In each plot, significant differences between non-outlier and outlier regions are marked with an asterisk next to the lineage name.

(a)

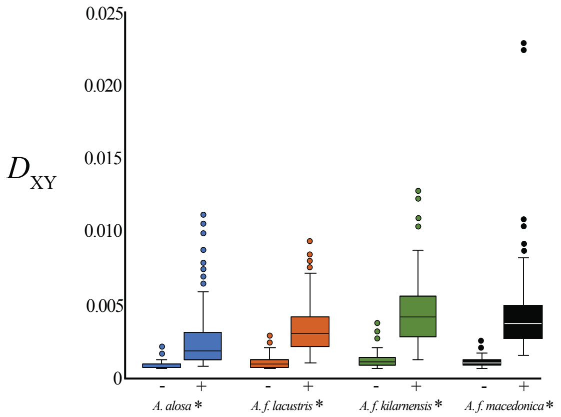

(b)

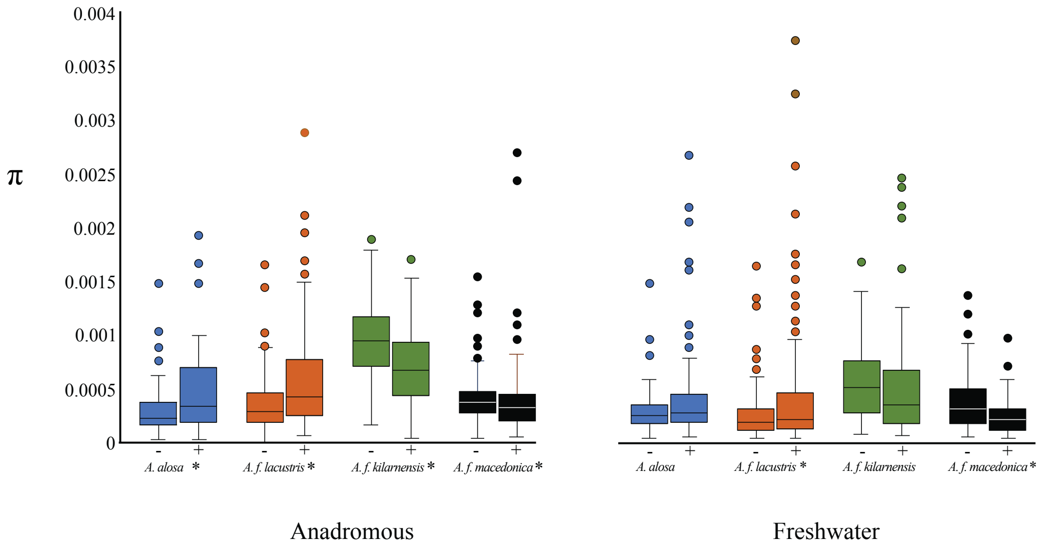

Supplemental Figure 7. Tajima's D (b) in non-outlier (-) and outlier (+) genomic regions in each of the 16 populations studied. Measurements are the averages for 20kb windows across each type of region. Populations are shown by numbered squares (anadromous) and circles (freshwater) as indicated in Figure 1.

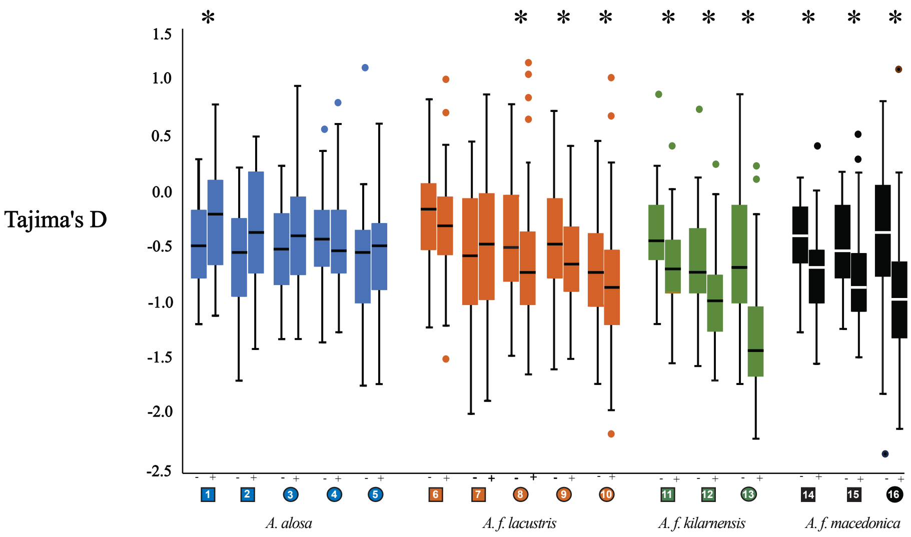

Supplemental Table 1. The sampling locations and size ("N") of the anadromous and freshwater populations of *Alosa* studied. The sequencing coverage for each pool of individuals per population is given under ("CV"). Each species/lineage is colored according to the map distance tree in Figure 1. The estimates of genetic diversity provided are average π for 20 kilobase genomic windows. Locations with while circles next to them are freshwater populations that do not migrate to marine waters.

Species	Sampling Location	N	CV	π	Country	Drainage Basin	Latitude	Longitude
A. alosa	Garonne River	48	30	0.00212	France	Atlantic	45°36'06"N	01°04'07"W
A. alosa	Mondego River	32	37	0.00199	Portugal	Atlantic	40°08'44"N	08°51'44"W
A. alosa ○	Aguieira Reservoir	29	36	0.00198	Portugal	Atlantic	40°21'10"N	08°10'30"W
A. alosa ○	Castelo de Bode Reservoir	21	27	0.00194	Portugal	Atlantic	39°32'41"N	08°18'57"W
A. alosa ○	Alqueva Reservoir	66	39	0.00182	Portugal	Atlantic	38°12'39"N	07°28'49"W
A. fallax	Lima River	19	29	0.00316	Portugal	Atlantic	41°41'09"N	08°49'57"W
A. fallax	Mondego River	11	33	0.00328	Portugal	Atlantic	40°08'44"N	08°51'44"W
A. f. killarnensis ○	Lough Leane	43	36	0.00263	Ireland	Atlantic	52°02'22"N	09°33'47"W
A. fallax	Tavignano River	15	24	0.00249	France	Mediterranean	42°06'13"N	09°32'58"E
A. fallax	Po River	20	26	0.00239	Italy	Mediterranean	44°57'12"N	12°25'56"E
A. f. lacustris ○	Lake Maggiore	14	28	0.0019	Italy	Mediterranean	45°59'02"N	08°40'29"E
A. f. lacustris ○	Lake Como	39	33	0.00194	Italy	Mediterranean	45°59'17"N	09°14'43"E
A. f. lacustris ○	Lake Garda	33	44	0.00198	Italy	Mediterranean	45°34'30"N	10°38'11"E
A. immaculata	Danube River - Tulcea	40	37	0.00279	Serbia	Black/Adriatic Seas	45°12'48"N	28°47'04"E
A. immaculata	Danube River - Iron Gate	25	26	0.00266	Romania	Black/Adriatic Seas	44°40'1"N	22°31'55"E
A. macedonica ○	Lake Volvi	12	31	0.00221	Greece	Black/Adriatic Seas	40°40'58"N	23°28'27"E

Supplemental Table 2. The Genbank accession number or gene model name of all sequences used for phylogenetic analysis of ATPase-α1 is provided.

ATPase		
Danio rerio - α1a1a	NM_131686.1	α1.1
Danio rerio - α1a.4	NM_131689.1	α1.1
Danio rerio - α1a.5	NM_178099.2	α1.1
Danio rerio - α1a.3	NM_131688.1	α1.1
Danio rerio - α1a.2	NM_131687.1	α1.1
Danio rerio - α1a.1b	NM_131690.1	α1.1
Alosa alosa - α1.1a	Allis_9072	α1.1
Alosa pseudoharengus - α1.1a	GFCK01198590.1	α1.1
Clupea harengus - α1.1a	XM_012815450.1	α1.1
Alosa pseudoharengus - α1.1b	GFCK01208951.1	α1.1
Alosa alosa - α1.1b	GETY01052870	α1.1
Alosa alosa - α1.2	Allis_124	α1.2
Danio rerio - α1.2a	NM_131683.1	α1.2
Danio rerio - α1.3b	AY008374.1	α1.3
Danio rerio - α1.3a	NM_131684.2	α1.3
Alosa alosa - α1.3	GETY01025548.1	α1.3
Clupea harengus - α1.3a	XM_012819956	α1.3
Clupea harengus - α1.3b	XM_012818286.1	α1.3
Xenopus tropicalis - nongastric-HKA	NM_001087349.1	nongastric
Mus musculus - nongastric-HKA	NM_138652.2	nongastric
Homo sapien - α1.4	GQ891529.1	α1.4
Mus musculus- gastric-HKA	NM_001290627.1	gastric
Xenopus tropicalis - gastric-HKA	NM_001090874.1	gastric

Supplemental Table 3. The Genbank accession numbers for the amino acid sequences used in the ATPase-α1 protein alignment are shown.

Species	Accession/Transcript
Annual killifish	XP_013874891
Mozambique tilapia	KC702514
Atlantic salmon	BT058747
Three-spined stickleback	BT027976
Atlantic herring	XP_012673740
Allis shad -Allele 1	GETY01052870
Allis shad -Allele 2	GETY01052870
Alewife	GFCK01208951
Milkfish	XP_030634863
W. Indian ocean coelacanth	XM_014491963
Japanese eel	KU976439
Spotted gar	XP_006639434.1
Spiny dogfish	2ZXE_A

Supplemental Table 4. Statistics on SNP loci and genomic windows for each of the four lineages studied. The number of bi-allelic SNP loci, the number of SNP loci used per genome window, the number of genome windows examined, and the average size of genome windows per lineage are provided.

	Number SNPs	SNP/ Win.	Interval (N. SNPs)	Number of Win.	Avg. Win. Size (bp)
A. alosa	1407776	20	10	139329	10833
A .f. lacustris	1581898	20	10	156753	9649
A .f. killarnensis	2187475	20	10	217296	6996
A. macedonica	1441886	20	10	142732	10573

Supplemental Table 5. Annotations for all outlier genomic windows in each lineage (See attached excel spreadsheet).

Supplemental Table 6. The number of outlier windows found using the ΔAF method and the number found using PCadapt. The percentage of outlier windows shared between the ΔAF method and PCadapt are shown.

	**Outlier Windows**			**ΔAF - PCadapt**	
	ΔAF		PCA		
	0.1	0.01	FDR	0.1	0.01
A. alosa	140	1,394	10,606	93%	85%
A. f. lacustris	157	1,568	7,618	89%	79%
A. f. killarnensis	218	2,173	6,409	75%	57%
A. macedonica	143	1,428	5,595	100%	89%

Supplemental Table 7. a) The number of shared polymorphic SNP loci between pairs of lineages and the average genetic distances (F_{ST}) between them. b) The shared number of SNP loci for the three naturally occurring lineages, and for all four studied. In each case, the percentage of polymorphism that is shared for each lineage is given in parentheses.

(a)

		Shared SNPs	F_{ST}
A. macedonica (4.3%)	*A. alosa* (4.4%)	61975	0.741
A. f. lacustris (6.5%)	*A. macedonica* (7.1%)	161406	0.707
A. f. lacustris (10.2%)	*A. alosa* (11.5%)	102105	0.746
A. macedonica (8.1%)	*A. f. killarnensis* (5.3%)	116340	0.648
A. alosa (29%)	A .f. killarnensis (18.7%)	408932	0.597
A. f. lacustris (26.3%)	*A. f. killarnensis* (19%)	415927	0.420

(b)

				Shared SNPs
A. alosa (4.92%)	*A. f. lacustris* (4.38%)	*A. f. killarnensis* (3.17%)		69241
A. alosa (1.41%)	*A. f. lacustris* (1.25%)	*A. macedonica* (1.37%)		19803
A. alosa (1.95%)	*A. f. killarnensis* (1.26%)	*A. macedonica* (1.91%)		27510
A. f. lacustris (4.38%)	*A. f. killarnensis* (3.17%)	*A. macedonica* (4.80%)		69241
A. alosa (0.62%)	*A. f. lacustris* (0.55%)	*A. f. killarnensis* (0.40%)	*A. macedonica* (0.61%)	8749

Supplemental Table 8. The outlier regions that overlapped among sets of three lineages in the first column are shown.

Lineages	Scaffold	Position	Genes
A. alosa / A. f. lacustris / A. macedonica	3	3147751:3153632	FOXO1
A. alosa / A. f. lacustris / A. f. killarnensis	1	3286046:3290803	ANKRD34A POLR3GL arrdc3
	43	2565039:2579150	
	171	201666:236147	nan bcap29 SO1C1 PDE3A
	411	131261:149447	axin AXIN1 GTF3C1
A. alosa / A. f. killarnensis / A. macedonica	57	2493888:2540054	S35B3 S35B3
	114	1002077:1027832	TC1 nan cna 5HT7R
A. f. lacustris / A. f. killarnensis / A. macedonica	8	6390750:6429471	ADAS pde5a
	29	2692135:2746969	mtnr1aa
	35	428534:450407	eif3d cuta sstr3 CYTH4
	48	1283295:1291952	Adap2 rhot1a C17orf75 znf207
	78	2523528:2523719	
	566	118694:159974	cbln5 C1Q

Supplemental Methods

RNAseq data for Cufflinks

We utilized four unpublished RNA sequence datasets generated in our lab to infer intro/exon boundaries for our *de novo A. alosa* genome annotation. These included data generated from RNA extracted from liver, gill, and muscle from *A. alosa*, and blood from *A. fallax*. In each case, tissue was dissected, or blood extracted, from individuals caught by local fishermen as they migrated upstream to spawn. The gill and muscle tissue were taken from the same individual caught in the Le Blavet River, France. And, the blood sample was taken from a single individual caught in marine waters off Espinho Portugal. Liver tissue, in contrast, was collected from 21 individuals caught in either the Guadiana or Minho Rivers in Portugal, or the Garonne River in France.

Tissues were diced into millimeter-sized pieces and stored at room temperature in 1.5mL vials filled with RNALater (Ambion) for several hours. The blood sample was extracted from the main artery of the individual caught using a syringe and then put in a vial with EDTA and shaken for five minutes. Subsequently, the blood sample was transferred into another vial containing RNALater. The RNA samples in RNAlater were stored at 4C for around one day and then transferred to -80C after the liquid RNALater was removed from the vial. Whole RNA was extracted from the gill tissue with the RNeasy Mini Kit (Qiagen), including a step to remove remaining DNA using RNase-Free DNase.

The liver RNA was sequenced as a pool of RNA from all 21 individuals, combined in equimolar ratios. The pooled RNA was then sequenced on a quarter of a plate of a 454 GS FLX Titanium sequencer at the Biocant Sequencing Facility in Cantanhede, Portugal. The resulting data was trimmed and quality filtered by the Biocant Sequencing team. The blood, gill, and muscle RNA samples were each used to make TruSeq RNA libraries (paired-end, 125 bp reads), which were then sequenced on an Illumina Hiseq1500. The RNA datasets were quality filtered using trimomatic which: (i) removed Illumina adapters, (ii) removed leading low-quality bases, (iii) removed low quality trailing bases, (iv) scanned reads with a sliding window of length 4 and cut when

the average quality dropped below 15 and (v) dropped reads of less than 50 bases in length.

Phylogenetic analysis of populations

Genetic relationships among the populations studied were examined using phylogenetic analysis as follows. Population allele frequencies were estimated for each population as outlined in the main text. We then randomly selected 10,000 SNP loci and used their allele frequencies to calculate pairwise F_{ST} (ANOVA method) using Poolfstat (https://cran.r-project.org/web/packages/poolfstat/poolfstat.pdf). Next, midpoint rooted trees were generated from the F_{ST} estimates using Phangorn (Schliep et al. 2017). This procedure was repeated 1000 times, and the final tree topology is based on a majority-rule consensus tree of the replicates. Branch lengths for the final tree were based on the average pairwise F_{ST} for all 1000 iterations. Statistical support for nodes in the final tree was calculated as the percentage of replicate trees with each node.

Population Genetics

Tajima's D and π were estimated with Popoolation using a sliding window approach with the following parameters: window size: 20 kb; step size: 5 kb; min-count: 2; min-qual: 20; pool-size: twice the number of individuals in the pool; min-coverage: 5; max-coverage: twice the genome-wide average; min-covered-fraction 0.2. For D_{XY}, to obtain the most accurate measure of minor allele frequencies (maf) possible with pool-seq data, we started by defining the ancestral state of all SNPs found as the most common state found in *Alosa sapidissima* (data not shown) and recomputing new pileups for all populations based on the new reference. Given that our data was now polarized to the most likely ancestral state, we used SNAPE-pooled (Raineri et al., 2012) to estimate the unfolded spectrum using the informative prior; theta: 0.001; D: 0.1 and nchr: twice the number of individuals in the pool. D_{XY} was then calculated using the resulting maf estimates with a script available in ngstools (https://github.com/mfumagalli/ngsTools: calcDxy.R). Statistical significance in all D_{XY}, Tajima's D, and π comparisons were accessed using a Mann-Whitney U test.

Supplemental Discussion

Here we further discuss the outlier regions identified in our study including the potential functions of genes found in them and their potential roles in adaptation in freshwater shad. We focus on genes and pathways that exhibited some degree of genetic convergence across lineages or populations in our study, as these are among the most likely to be true targets of natural selection. Our goal here is just to provide some additional support for our conclusions about parallelism made in the main paper and guide future research.

In our investigation, we identified two large (greater than 100 kilobases) outlier regions that contained multiple genes. In the main paper, we analyze and discuss one such region found in scaffold 411 that contained *ATPase-α1.1*b, including nonsynonymous mutations in the gene that had strong ΔAF between anadromous and freshwater populations of either or both *A. alosa* and *A. f. lacustris*. Most research on the role of *ATPase-α1* in adaptation in fish species highlights the importance of the expression of the gene in gill and/or gut tissue for osmoregulation (e.g., (Sáez et al. 2009; Wong et al. 2016) and references therein). Our results indicate that protein-coding changes may also be important in this regard. Other genes included in the same outlier region that contained *ATPase-α1.1*b were *Mkr2*, *Thumpd1*, *Axin1-like*, *GTF3C1*, *sec1411*, and PPuP5343. When considering the consistency of extreme ΔAF across populations, and their magnitude, together, the most likely candidate gene in this outlier region in *A. alosa* was not *ATPase-α1.1*b, but *Axin1-like* (Supplemental Figure 2A). This is true because the highest ΔAF for this region in one of the freshwater populations of *A. alosa* studied, the Aguieira, were those that contained *Axin1-like* and not *ATPase-α1.1*b. The highest ΔAF for *Axin1-like* in the Aguieira were concentrated around a nonsynonymous mutation that also exhibited extreme allele frequency shifts in the other two freshwater populations of *A. alosa* that we examined (the Castelo de Bode and Alqueva). As discussed in the main text, this nonsynonymous mutation in *Axin1-like* was linked with three nonsynonymous mutations found in *ATPase-α1.1*b, all of which exhibited extreme ΔAF between freshwater and anadromous *A. alosa*. These results suggest that *Axin1-like*

may be the main target of positive selection in the Aguieira, while in Castelo de Bode and Alqueva, it is both *Axin1-like* and *ATPase-α1.1*b. The function of the ion-transport gene *ATPase-α1.1*b is discussed in the main test. *Axin1* is part of the Wnt signaling pathway, which is an intercellular signaling network conserved across invertebrates and vertebrates that plays important roles in tissue growth, polarity, and patterning (Swarup and Verheyen 2012). Knocking out *Axin1* in zebrafish results in loss of cerebral asymmetry that causes reduced motility and loss of responsiveness to either visual or olfactory stimuli (Carl et al. 2007; Dreosti et al. 2014) Given what is known about the function of *Axin1* in other fish species, it is possible that *Axin1-like* is involved in life history changes related to behavior, such as migration (i.e. swimming capacity or navigation) or feeding ecology in freshwater *A. alosa*. That nonsynonymous mutations within *ATPase-α1.1*b and *Axin1-like* were found to be co-segregating in freshwater populations of *A. alosa* suggests they may be evolving in concert as a coadapted gene complex.

Another larger outlier genomic region found in more than one lineage in our study was a ~300kb region in *A. f. killarnensis* and *A. macedonica* that contained *Piezo2*, *Impa2*, *mppe1*, *gnal*, *Elov2*, *gcm2* and *mak* (Supplemental Figure 1). The areas within this genomic region that overlapped the most between *A. f. killarnensis* and *A. macedonica* where those that contained or were nearby to *Piezo2, Impa2*, *mppe1*, and *Elov2*, making these especially good candidate genes. Nonsynonymous mutations with significant differences in frequencies between anadromous and freshwater populations were found for these genes in one (*mppe1*, and *Elovl2)* or both (*Piezo2*) *A. f. killarnensis* and *A. macedonica*, but none of these changes were shared between the two lineages. This subset of candidate genes has a variety of biological functions. In tilapia (Gardell et al. 2013; Wang and Kültz 2017), eel (Kalujnaia et al. 2016), and zebrafish, *Impa2*, which is part of the myo-inositol biosynthetic pathway, plays a central role in adaptation to osmotic stress. *Elov2* is involved in the biosynthesis of long-chain polyunsaturated fatty acids and is crucial in many genetic pathways including those involved in metabolism (Monroig et al. 2016) to aging of the retina (Chen et al.). In zebrafish larvae, *Elov2* is significantly down-regulated when they are exposed to hypoxic and cold conditions (Long et al. 2015). The function of *mppe1* is not well studied in many fish species, but plays a role in cellular transport and lipid remodeling in vertebrates

(http://www.xenbase.org/). *Piezo2* is often involved in mechanoreception (Moroni et al. 2018) and, for example in salmon, likely plays a role in the sensing water flow via its adipose fin (Koll et al. 2020). With perhaps the exception of *mppe1*, all of the candidate genes in this region are known to have specific biological functions in fish species that each could plausibly affect the fitness of freshwater shad.

Several candidate genes we identified have functions related to neuronal activity, eyesight, metabolism, stress response and behavior including migration, and reproduction. For example, *5ht7r*, an outlier in *A. alosa* and *A. macedonia*, is a serotonin receptor that is a well-known modifier of behavior and reproduction in fish species (Maximino et al. 2013; Prasad et al. 2015), but may also play a role in responding to hypoxia (Panlilio et al. 2016). The gene found in the candidate region with the highest ΔAF across all three populations of *A. alosa* studied was *Vgll4*. The genomic region containing *Vgll4* in *A. alosa* had the second highest ΔAF for the lineage, overall, which resulted from strong allele frequency shifts in SNPs upstream of the gene in all three freshwater populations we examined (Supplemental Figure 3). A paralog of *Vgll4*, *Vgll3*, was shown to control the age at maturity in Atlantic salmon, *Salmo salar* (Barson et al. 2015), which is a trait that is central to most anadromous life histories. However, in zebrafish, *Vgll4* is mainly expressed in the otic vesicle and pectoral fins during development (Xue et al. 2018), suggesting it may play a role in movement, sight, or behavior. Another study showed that *Vgll4b* mutant zebrafish had an impaired erythroid phenotype that affected erythropoiesis terminal differentiation and their capacity to handle hypoxia (Wang et al. 2019). The most extreme outlier region in *A. f. killarnensis* contained two genes, *cyp17a* and *fbxo-41*. The cytochrome-oxidase gene, *cyp17a*, is crucial for gonadal development and plays a key role in reproduction in fish species (Hinfray et al. 2013). The role of *fbxo-41* is known to be involved in brain development in vertebrates (King et al. 2019). Based on what is known about the functions of these candidate genes, the transition from an anadromous to freshwater life history in Alosa may involve significant changes in the brain and eye development, and phenotypic and hormonal changes related to stress response, reproduction, and migratory behavior.

In addition to the ion-transport gene *ATPase-α1.1*b, which has already been discussed, two other ATPase genes were identified as candidates in our study. One of

them, *ATPase-* β, was a candidate gene found in *A. f. killarnensis*. While the functional role of *ATPase-* α *1* in many fish species is well established, that of ATPase- β is not well understood (Rajarao et al. 2001) but is expected to be distinct from that of *ATPase-* α, possibly modifying ion-transport function (Hilbers et al. 2016). The other candidate ATPase gene identified in our study was *ATPase-* α *1.3* in *A. f. lacustris*. *ATPase-* α *1.3* is an ion-transport gene that is expressed in the gut, eye, testis, and brain in zebrafish (Rajarao et al. 2001) and is known for its role in neuron function in mammals (Wong et al. 2016). Knockouts of *ATPase-* α *1.3 in* zebrafish embryos result in brain ventricle dilation, probably due to acute ionic imbalances (Doğanlı et al. 2013) (Doganli et al. 2013). Yet, interestingly, based on gene expression analysis, *ATPase-* α *1.3* does have salinity sensitivity in tilapia and killifish, but not stickleback (Wong et al. 2016).

References

Barson NJ, Aykanat T, Hindar K, Baranski M, Bolstad GH, Fiske P, Jacq C, Jensen AJ, Johnston SE, Karlsson S, et al. 2015. Sex-dependent dominance at a single locus maintains variation in age at maturity in salmon. *Nature* 528:405–408.

Carl M, Bianco IH, Bajoghli B, Aghaallaei N, Czerny T, Wilson SW. 2007. Wnt/Axin1/Î²-Catenin Signaling Regulates Asymmetric Nodal Activation, Elaboration, and Concordance of CNS Asymmetries. 55:393–405.

Chen D, Chao DL, Rocha L, Aging MK, 2019. The Lipid Elongation Enzyme ELOVL2 is a molecular regulator of aging in the retina. *Wiley Online Libr.* [Internet]. Available from: https://onlinelibrary.wiley.com/doi/abs/10.1111/acel.13100

Doğanlı C, Beck HC, Ribera AB, Oxvig C, Lykke-Hartmann K. 2013. α3Na+/K+-ATPase Deficiency Causes Brain Ventricle Dilation and Abrupt Embryonic Motility in Zebrafish. *J. Biol. Chem.* 288:8862–8874.

Dreosti E, Llopis NV, Carl M, Yaksi E, Wilson SW. 2014. Left-Right Asymmetry Is Required for the Habenulae to Respond to Both Visual and Olfactory Stimuli. *Curr. Biol. CB* 24:440–445.

Gardell AM, Yang J, Sacchi R, Fangue NA, Hammock BD, Kultz D. 2013. Tilapia (*Oreochromis mossambicus*) brain cells respond to hyperosmotic challenge by inducing myo-inositol biosynthesis. *J. Exp. Biol.* 216:4615–4625.

Hilbers F, Kopec W, Isaksen TJ, Holm TH, Lykke-Hartmann K, Nissen P, Khandelia H, Poulsen H. 2016. Tuning of the Na,K-ATPase by the beta subunit. *Sci. Rep.* 6:1–11.

Hinfray N, Nóbrega RH, Caulier M, Baudiffier D, Maillot-Maréchal E, Chadili E, Palluel O, Porcher J-M, Schulz R, Brion F. 2013. Cyp17a1 and Cyp19a1 in the zebrafish testis are differentially affected by oestradiol. *J. Endocrinol.* 216:375–388.

King CR, A A Quadros AR, Chazeau A, Saarloos I, van der Graaf AJ, Verhage M, Toonen RF. 2019. Fbxo41 Promotes Disassembly of Neuronal Primary Cilia. *Sci. Rep.* 9:8179.

Koll R, Martorell Ribera J, Brunner RM, Rebl A, Goldammer T. 2020. Gene Profiling in the Adipose Fin of Salmonid Fishes Supports Its Function as a Flow Sensor. *Genes* 11:21–22.

Long Y, Yan J, Song G, Li Xiaohui, Li Xixi, Li Q, Cui Z. 2015. Transcriptional events co-regulated by hypoxia and cold stresses in Zebrafish larvae. *BMC Genomics*. Available from: https://doi.org/10.1186/s12864-015-1560-y

Maximino C, Puty B, Benzecry R, Araújo J, Lima MG, de Jesus Oliveira Batista E, de Matos Oliveira KR, Crespo-Lopez ME, Herculano AM. 2013. Role of serotonin in zebrafish (Danio rerio) anxiety: Relationship with serotonin levels and effect of buspirone, WAY 100635, SB 224289, fluoxetine and para-chlorophenylalanine (pCPA) in two behavioral models. *Neuropharmacology* 71:83–97.

Monroig Ó, Lopes-Marques M, Navarro JC, Hontoria F, Ruivo R, Santos MM, Venkatesh B, Tocher DR, Castro LFC. 2016. Evolutionary functional elaboration of the Elovl2/5 gene family in chordates. *Sci. Rep.* 6:20510–10.

Moroni M, Servin-Vences MR, Fleischer R, Sánchez-Carranza O, Lewin GR. 2018. Voltage gating of mechanosensitive PIEZO channels. *Nat. Commun.* 9:1096–15.

Panlilio JM, Marin S, Lobl MB, McDonald MD. 2016. Treatment with the selective serotonin reuptake inhibitor, fluoxetine, attenuates the fish hypoxia response. *Sci. Rep.* [Internet]. Available from: http://dx.doi.org/10.1038/srep31148

Prasad P, Ogawa S, Parhar IS. 2015. Role of serotonin in fish reproduction. *Front. Neurosci.* 9:328–329.

Raineri, E., Ferretti, L., Esteve-Codina, A., Nevado, B., Heath, S., & Pérez-Enciso, M. (2012). SNP calling by sequencing pooled samples. *BMC Bioinformatics*, *13*(1), 239. https://doi.org/10.1186/1471-2105-13-239

Rajarao SJ, Canfield VA, Mohideen MA, Yan YL, Postlethwait JH, Cheng KC, Levenson R. 2001. The repertoire of Na,K-ATPase alpha and beta subunit genes expressed in the zebrafish, Danio rerio. *Genome Res.* 11:1211–1220.

Sáez AG, Lozano E, Zaldívar-Riverón A. 2009. Evolutionary history of Na,K-ATPases and their osmoregulatory role. *Genetica* 136:479–490.

Schliep K, Potts AJ, Morrison DA, Grimm GW. 2017. Intertwining phylogenetic trees and networks.Fitzjohn R, editor. *Methods Ecol. Evol.* 8:1212–1220.

Swarup S, Verheyen EM. 2012. Wnt/Wingless Signaling in Drosophila. *Cold Spring Harb. Perspect. Biol.* [Internet] 4:a007930. Available from: http://cshperspectives.cshlp.org/lookup/doi/10.1101/cshperspect.a007930

Wang X, Kültz D. 2017. Osmolality/salinity-responsive enhancers (OSREs) control induction of osmoprotective genes in euryhaline fish. *Proc. Natl. Acad. Sci.*:201614712–10.

Wang Y, Liu X, Xie B, Yuan H, Zhang Y, Zhu J. 2019. The NOTCH1-dependent HIF1α/VGLL4/IRF2BP2 oxygen sensing pathway triggers erythropoiesis terminal differentiation. *Redox Biol.* [Internet] 28. Available from: https://doi.org/10.1016/j.redox.2019.101313

Wong MK-S, Pipil S, Ozaki H, Suzuki Y, Iwasaki W, Takei Y. 2016. Flexible selection of diversified Na+/K+-ATPase α-subunit isoforms for osmoregulation in teleosts. *Zool. Lett.* 2:1–22.

Xue C, Wang HH, Zhu J, Zhou J. 2018. The expression patterns of vestigial like family member 4 genes in zebrafish embryogenesis. *Gene Expr. Patterns GEP* 28:34–41.
